# Identification and Characterization of the PEBP Family Genes in Moso Bamboo (*Phyllostachys heterocycla*)

**DOI:** 10.1038/s41598-019-51278-7

**Published:** 2019-10-18

**Authors:** Zhaohe Yang, Lei Chen, Markus V. Kohnen, Bei Xiong, Xi Zhen, Jiakai Liao, Yoshito Oka, Qiang Zhu, Lianfeng Gu, Chentao Lin, Bobin Liu

**Affiliations:** 1 College of Forestry, Fujian Agriculture and Forestry University, Fuzhou, 350002 Fujian China; 2 Basic Forestry and Proteomics Research Center, Fujian Agriculture and Forestry University, Fuzhou, 350002 Fujian China; 3 College of Life Science, Fujian Agriculture and Forestry University, Fuzhou, 350002 Fujian China; 4 Department of Molecular, Cell & Developmental Biology, University of California, Los Angeles, CA 90095 USA

**Keywords:** Plant breeding, Plant molecular biology

## Abstract

Moso bamboo is one of the economically most important plants in China. Moso bamboo is a monocarpic perennial that exhibits poor and slow germination. Thus, the flowering often causes destruction of moso bamboo forestry. However, how control of flowering and seed germination are regulated in moso bamboo is largely unclear. In this study, we identified 5 members (PhFT1-5) of the phosphatidyl ethanolamine-binding proteins (PEBP) family from moso bamboo genome that regulate flowering, flower architecture and germination, and characterized the function of these PEBP family genes further in Arabidopsis. Phylogenetic analysis revealed that 3 (PhFT1, PhFT2 and PhFT3), 1 (PhFT4) and 1 (PhFT5) members belong to the TFL1-like clade, FT-like clade, and MFT-like clade, respectively. These PEBP family genes possess all structure necessary for PEBP gene function. The ectopic overexpression of PhFT4 and PhFT5 promotes flowering time in Arabidopsis, and that of PhFT1, PhFT2 and PhFT3 suppresses it. In addition, the overexpression of PhFT5 promotes seed germination rate. Interestingly, the overexpression of PhFT1 suppressed seed germination rate in Arabidopsis. The expression of PhFT1 and PhFT5 is significantly higher in seed than in tissues including leaf and shoot apical meristem, implying their function in seed germination. Taken together, our results suggested that the PEBP family genes play important roles as regulators of flowering and seed germination in moso bamboo and thereby are necessary for the sustainability of moso bamboo forest.

## Introduction

Bamboos are economically and ecologically important plant species with high competition ability of biomass yield due to fast growth^1,2^. Moso bamboo (*Phyllostachys heterocycla*) is one of the most important bamboo species in China, because the moso bamboo forests cover about 6 million hectare representing 3% of the total Chinese forest area and the total annual production of moso bamboo forest was valued at five billion US dollars in 2013^3^. Bamboos are monocarpic perennial plants that live for decades or even longer before flowering a single time, set seeds and die^3,4^. Because of its gregarious and monocarpic nature, flowering often results in the bamboo forest degradation leading to the economic and ecological loss^5^.

The transition from the vegetative to reproductive phase is a critical event in the life cycle of plants and their survival as species especially in bamboo. Although in large bamboo plantations the clonal propagation is more impactful than the sexual reproduction due to limitation of seeds harvest, preservation and low germination rate, it is a high cost and low efficiency propagation way for bamboo plantations since the requirement of adequate reserves of bamboo tissue, destruction of bamboo forestry and lack of convenient transportation^6^. Furthermore, seed dormancy and seed germination have also a considerable impact on the reproduction of moso bamboo forests, because seeds often germinate poorly and slowly which can take up to months or even years^7,8^. Across all three major evolutionary lineages the phosphatidyl ethanolamine-binding protein (PEBP) gene family plays a pivotal role in a variety of biological processes including the regulation of floral transition and seed germination^9–12^.

The plant PEBP family can be classified into three subfamilies: FLOWERING LOCUS T (FT)-like, TERMINAL FLOWER1 (TFL1) -like and MOTHER OF FT AND TFL1 (MFT) -like clades. The functions of PEBP family genes have been extensively analyzed in *Arabidopsis thaliana* (Arabidopsis). The AtFT-like subfamily comprises two genes, *FT* and *TWIN SISTER OF FT* (*TSF*)^13^. The FT protein acts as a florigen, a systemic signaling molecule that promotes flowering^14,15^. Namely, FT protein expression is induced in leaves in response to day length and transported in sieve element to the shoot apical meristem (SAM)^16–19^. In the SAM, FT interacts with FLOWERING LOCUS D (FD), a basic leucine zipper domain transcription factor, to activate downstream signaling components and complete floral transition^20^. Unlike FT-like proteins, members of the TFL1-like family inhibit flowering by competing with FT to regulate FD function^21–23^. The Arabidopsis genome encodes three TFL1-like genes, *TFL1*, *BROTHER OF FT AND TFL1* (*BFT*) and *ARABIDOPSIS THALIANA CENTRORADIALIS* (*ATC*). *TFL1* and *BFT* genes are expressed in inflorescence meristems and delay the flowering time though negatively regulating its development^24^. In contrast, *ATC* gene expression has only been shown in hypocotyls of young seedlings^22^. Accordingly, the loss-of-function mutant of *ATC* does not show a flowering and inflorescence architecture phenotype^22^. However, constitutively expressed ATC showed a weak capacity to complement early flowering and terminal flower formation of *terminal flower 1*-*1* (*tfl1*-*1*) mutant phenotypes. Its overexpression in wild type plants exhibited late flowering phenotype and aberrant inflorescence architecture, suggesting that ATC protein has similar function to that of TFL1 and BFT^22,23^. MFT-like genes are the evolutionary ancestor of the FT-like and TFL-like genes. MFT has weak activity to promote flowering in Arabidopsis^25^. In addition, it has been recently reported that MFT mediates seed germination^26^. The mechanism, by which MFT regulates seed germination, is largely elusive, but it regulates the expression of key genes in the regulation of seed germination^26^.

PEBP proteins are characterized by the presence of two highly conserved short motifs, DPDxP and GxHR, which presumably contribute to the conformation of the ligand binding pocket^12,27^. In Arabidopsis FT and TFL1 proteins are 175 and 177 amino acids long, respectively^14^. Despite their opposite function on flowering both proteins contain only 39 nonconserved amino acids^28^. Interestingly, substitution of the single amino acid, Tyr^85^ to His, in FT partially converts FT function to TFL1 function probably though discrimination of structurally related interactors^28^. In addition, the amino acid sequence encoded by the the fourth exon plays a critical role to determine FT and TFL1 protein functions and can be divided into four segments (segment A–D) by sequence conservation^29^. Segment B and segment C containing the LYN/IYN triplet conserved motif are especially important for the determination of functional specificity between FT and TFL1^29^. In sugar beet two FT paralogs with antagonistic function have been identified^30^. BvFT1 and BvFT2 vary in segment B in the three amino acids, Tyr^138^, Gly^141^ and Trp^142^, and exon swapping successfully changed promoting and repressing activity^30^.

The PEBP family genes appear to play similar roles in other plant species. Specifically, the rice FT-like gene, *HEADING DATE3A* (*Hd3a*), is up-regulated in response to short day, but not in long day condition, and the overexpression of *Hd3a* leads to the early-heading phenotype^31^. The overexpression of rice TFL1-like genes, either *Rice TERMINAL FLOWER 1*/*CENTRORADIALIS 1* (*RCN1*) or *RCN2*, results in late flowering phenotype and abnormal panicle morphology in rice^32^. In addition, heterologous expression analyses, in which the PEBP family genes from tomato^33^, orchid^34^, Japanese apricot^35^, rubber tree^36^ and physic nut^37^ were expressed mostly in Arabidopsis, revealed that FT-like genes and TFL1-like genes promote and repress flowering, respectively. And MFT-like genes were involved in seed germination^26^. In other bamboo species, PEBP family genes were identified and characterize from *Phylostachys meyeri*^38^, *Shibataea chinensis*^39^, *Dendrocalamus latiforus*^40^, *Bambusa tulda*^41^ and *Phylostachys heterocycla*^1^. Specifically, two FT-like genes, PvFT1 and PvFT2 from *Phyllostachys violascens* are involved in promoting bamboo flower and development of floral organs, respectively^42^. BoTFL1-like from *Bambusa oldhamii* paly an inhibitor role of flowering^43^. These analyses revealed that TFL1-like genes regulate flower architecture and MFT-like genes promote seed germination.

Despite of extensive efforts, the mechanism underlying bamboo reproduction is largely unknown, mainly due to the long-term unflowered status and the lack of efficient transformation system. The completion of moso bamboo genome project identified several PEBP family genes as well as orthologues of FD and floral identity genes, suggesting that PEBP family genes regulate moso bamboo flowering. However, none of moso bamboo PEBP genes has been functionally analyzed, although some of PEBP family genes from other bamboo species have been tested. In this study, we examined the expression of 5 PEBP family genes from moso bamboo and functionally analyze their heterologous expression in Arabidopsis. Our results suggest the potential involvement of PEBP family genes in moso bamboo flowering and seed germination.

## Results

### Isolation and identification of PEBP family genes in moso bamboo

To identify PEBP proteins in moso bamboo we blast screened the entire moso bamboo genome database (http://server.ncgr.ac.cn/bamboo/blast.php) for genes providing sequence similarity with Arabidopsis and rice PEBP proteins. We obtained 6 PEBP family candidate genes from moso bamboo genome, but PH01000020G1780 were excluded from PEBP family because of harboring an incomplete PEBP domain with a lower expectation value (E = 7.8e-8). Therefore, 5 full-length PEBP family genes were identified and designated as *PhFT1*-*5* (Table S1),that were consistent with previously reported sequence^1^. In order to confirm this results, we cloned the full-length coding sequence of *PhFT1*, *PhFT2*, *PhFT3*, *PhFT4* and *PhFT5* from cDNA extracted from moso bamboo seedling.

**Figure 1:**
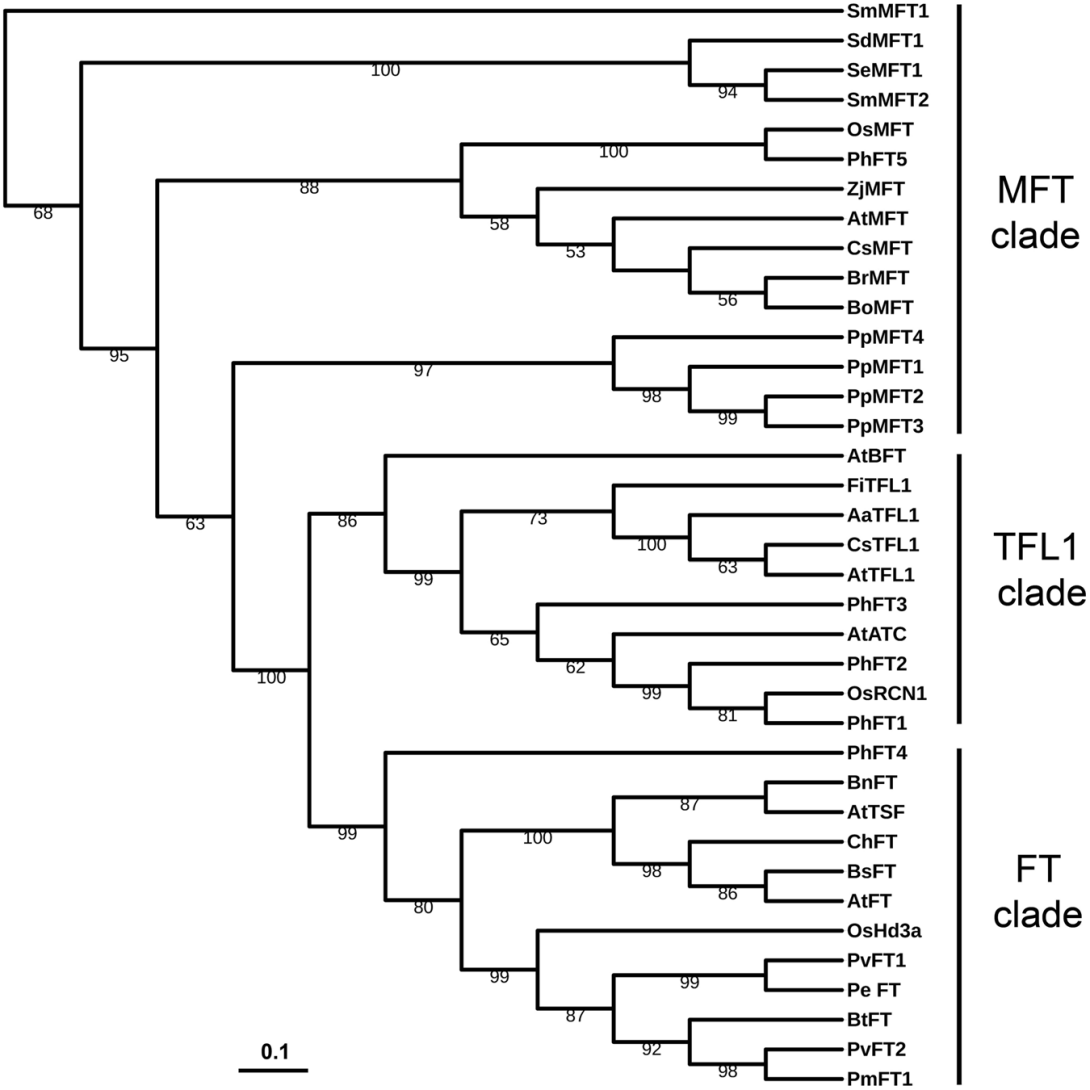
Comparative analysis of PEBP family proteins from moso bamboo and other plants. The phylogenetic tree of PEBP proteins from *Phyllostachys heterocycla* (Ph), *Arabidopsis thaliana* (At), *Oryza sativa* (Os), *Selaginella erythropus* (Se), *Camelina sativa* (Cs), *Brassica rapa* (Br), *Brassica oleracea* (Bo), *Ziziphus jujuba* (Zj), *Cardamine hirsuta* (Ch), *Boechera stricta* (Bs), *Brassica napus* (Bn), *Camelina sativa* (Cs), *Arabis alpina* (Aa), *Fragaria iinumae* (Fi), *Phyllostachys violascens* (Pv), *Selaginella moellendorffii* (Sm), *Selaginella denticulata* (Sd), *Bambusa tulda* (Bt), *Phyllostachys edulis* (Pe) and *Phyllostachys meyeri* (Pm) was constructed by IQ-TREE 1.6.9^84^. The unit for the scale bar displays branch lengths.

To future analyze the phylogenetic relationships between PEBP family proteins of moso bamboo and other species, we generated an rooted phylogenetic tree based on the full length PEBP protein sequences from *Phylostachys heterocycla*, *Arabidopsis thaliana*, *Oryza sativa*, *Selaginella erythropus*, *Camelina sativa*, *Brassica rapa*, *Brassica oleracea*, *Ziziphus jujuba*, *Cardamine hirsuta*, *Boechera stricta*, *Brassica napus*, *Camelina sativa*, *Arabis alpina*, *Fragaria iinumae*, *Phyllostachys violascens*, *Selaginella moellendorffii*, *Selaginella denticulata*, *Bambusa tulda*, *Phyllostachys edulis* and *Phyllostachys meyeri*^11,42,43^. According to the phylogenetic tree, the 5 PEBP family protein sequences from moso bamboo fall into three branches: MFT-like clade, which only contained PhFT5; TFL-like clade, which included PhFT1, PhFT2 and PhFT3; and FT-like clade, which contained PhFT4 (Fig. 1). Among these, PhFT5 shared 66.7% and 88.1% amino acid sequence identities to Arabidopsis and rice MFT, respectively (Table S1). PhFT1, PhFT2 and PhFT3 had 71.51%, 70.39% and 71.35% amino acid sequence identity to Arabidopsis TFL1 and 98.27%, 96.53% and 83.24% to rice RCN1, respectively (Table S1). PhFT4 shared 61.58% similar amino acids to Arabidopsis FT and 60% to rice Hd3a (Table S1).

**Figure 2:**
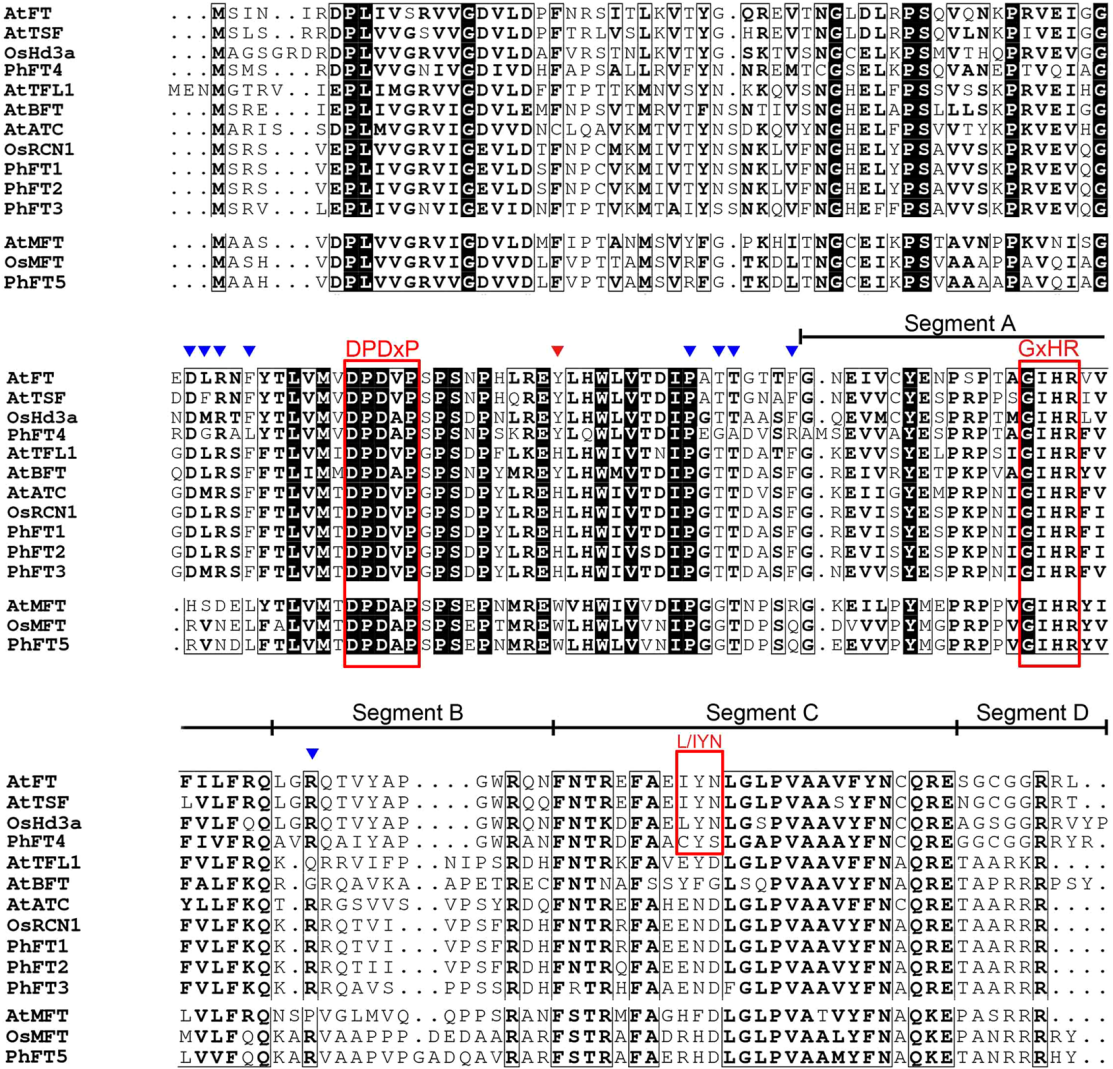
Sequence comparison of PEBP family proteins. Amino acid alignment of PEBP family proteins from *Phyllostachys heterocycla* (Ph) *A*. *thaliana* (At), *Oryza sativa* (Os) is shown. The red triangle indicates a key amino acid residue that determines FT-like and TFL1-like functions. The blue triangles indicate amino acid residues that interact with 14-3-3 protein. Red boxes represent the conserved DPDxP, GxHR motif and L/IYN motif, respectively. Underlines represent segment A, B, C and D, respectively.

Furthermore, the multiple protein sequences alignment revealed that moso bamboo PEBP family proteins have conserved PEBP domain and DPDxP motif (Fig. 2). The key amino acid residues that are distinguishable among the MFT-like (W), TFL-like (H) and FT-like (Y) clade were present at position 85 of AtFT in each moso bamboo PEBP family proteins (Fig. 2). However, the highly conserved amino acid sequences, LGRQTVYAPGWRQN in segment B and LYN triad in segment C are less conserved in PhFT4, although these motifs are determinant of FT activity and FT/TFL1 function (Fig. 2). Notably, these motives in PhFT4 are even different from FT sequences of other bamboo species (Fig. S1). Taken together, MFT-like and TFL-like clade of moso bamboo were conserved across angiosperm species, but FT-like clade is more diversified.

### The developmental stage dependent PhPEBPs expression in leaf and flowering tissue in moso bamboo

**Figure 3:**
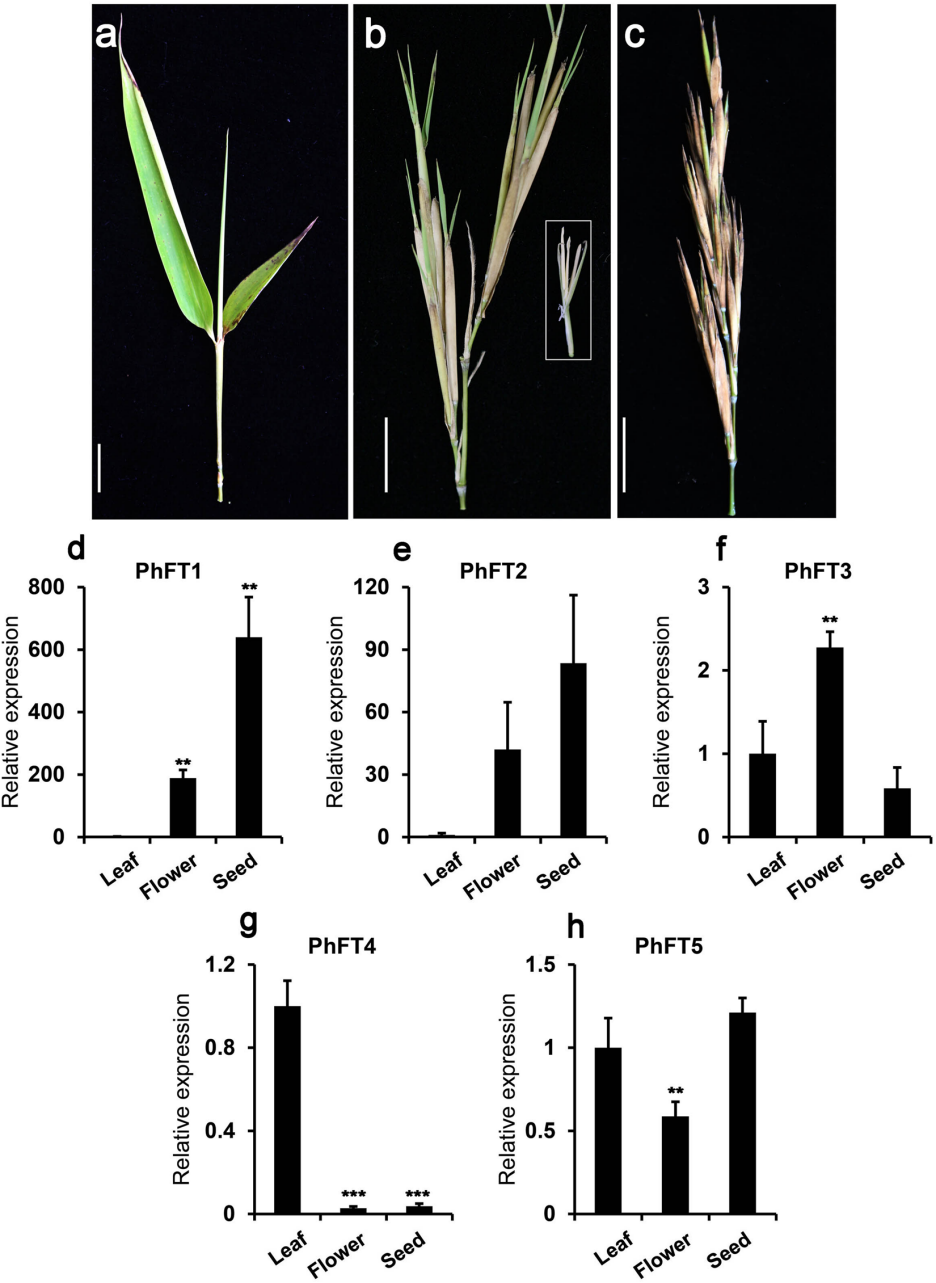
Expression pattern of *PhPEBP*s in flowering processes of moso bamboo. (**a**–**c**) Images showing apical region of moso bamboo leaf before flowering (**a**) flower and sheath at bloom (**b**) and seed sample after bloom (**c**). Inset in (**b**) showed the flower of moso bamboo. Scale bar = 20 mm. (**d**–**i**) qPCR analysis showing dynamic changes of *PhPEBPs* genes expression during moso bamboo flower development. The qPCR signals of each *PhPEBP* leaf, flowering tissue and sheath or seed were normalized with those of *PhUBQ* (Table S2) at corresponding time. The relative expression was calculated with the normalized qPCR signal at leaf before flowering set to 1. Values from three biological replicates are plotted with SE. ** and *** denote statistically significant differences determined by Student’s t test (P < 0.01) and (P < 0.001), respectively.

To gain insights into possible roles of these PEBP family genes in the regulation of flowering and/or flower development in moso bamboo, the expression pattern of PEBP family genes in the different developmental stages were analyzed. We could successfully obtain the samples from the flowering bamboo forest at Nanping, Fujian, China (Fig. S2). During the flowering of moso bamboo, the leaves began to die gradually (Fig. S2). Therefore, for RNA extraction leaf blades before flowering (leaf), leaf sheath and flowering tissues at bloom (flower) and developing seeds after bloom (seed) were sampled (Fig. 3a–c) and gene expression was subsequently tested by qRT-PCR analysis (Fig. 3d–h).

The expression of genes in the TFL-like clade had highly different transcript levels during the developmental stages investigated. Expression of *PhFT1* and *PhFT2* followd a similar pattern over time and tissue with a higher transcriptional abundance of *PhFT1* compared to *PhFT2*. No expression was detected in leaves prior to flowering, while during blooming *PhFT1* and *PhFT2* were clearly expressed in floral tissues and showed high transcript levels in developing seeds (Fig. 3d,e). *PhFT3* expression was also higher at blooming than in leaves before flowering. During seed development expression was comparably low with our leaves samples (Fig. 3f). By contrast, *PhFT4* was expressed in leaves prior flowering, however, hardly detectable in the other two samples (Fig. 3g). The expression of *PhFT5* was constant between the tested tissues and developmental stages, although *PhFT5* expression was slightly lower at blooming, (Fig. 3h).

### Ectopic expression of PhPEBPs in transgenic arabidopsis plants

In order to examine the function of moso bamboo PEBP family genes, fusion constructs of *PhPEBP* coding sequences and yellow fluorescence protein (YFP) driven by the *CaMV 35S* promoter were introduced into *Arabidopsis thaliana* wild type. We successfully obtained multiple transgenic lines for all constructs. The microscopic observation with these lines revealed that moso bamboo PEBP proteins localize in both of nucleus and cytoplasm in Arabidopsis (Fig. S3). These results are consistent with the observation that PEBP proteins from other plant species localize in both of nucleus and cytoplasm^20^.

**Figure 4:**
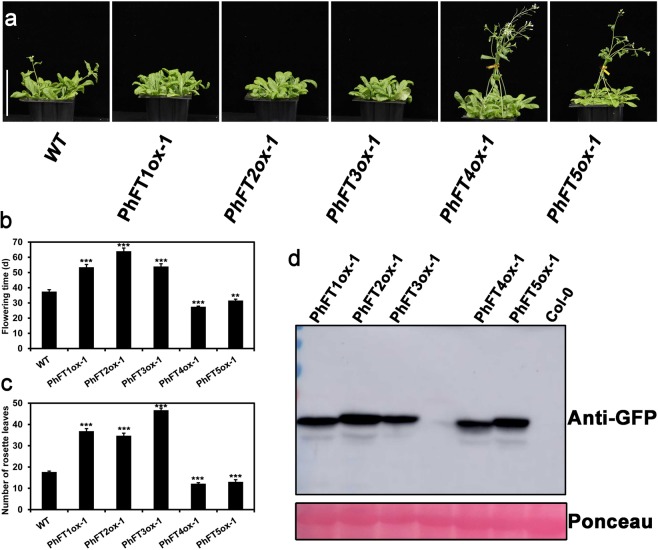
Overexpression of PhPEBPs alter flowering time in Arabidopsis. (**a**) Images of indicated genotypes grown in long day condition (16 hours light, 8 hours dark) for 38 days. Scale bar = 50 mm. (**b**,**c**) The time to flowering (**b**) and the number of rosette leaves at the time of flowering (**c**) of indicated genotypes grown in long day condition are shown with standard deviations (SD, n > 20). (**d**) Immunoblot detection of PhPEBPs proteins in indicated PhPEBPs overexpressors. Blots were stained with ponceau-S staining solution to confirm equal loading (lower panel), de-stained and probed with anti-GFP antibody (upper panel). Full scan data of immunoblot shown in Supplementary Fig. 13. ** and *** denote statistically significant differences determined by Student’s t test (P < 0.01) and (P < 0.001), respectively.

To examine the function of PhPEBPs in the floral regulation, we compared the flowering time between wild type and PhPEBPs-YFP overexpressing lines. As showed in Fig. 4a–c, wild type Col-0 flowered at 34 ± 2 days after germination with 18 ± 1.1 rosette leaves under long day condition. In this condition, the overexpressors of *PhFT1*-*YFP*, *PhFT2*-*YFP* and *PhFT3*-*YFP* flowered later at 46.5 ± 1.2, 68 ± 6.8 and 70 ± 1.2 days after germination with 35.5 ± 1.2, 34.5 ± 3.6 and 47.5 ± 2.0 rosette leaves, respectively (Fig. 4b,c). The late flowering phenotype of *PhFT1*-*YFP*, *PhFT2*-*YFP* and *PhFT3*-*YFP* roughly correlated with their expression level (Fig. S4). By contrast, the overexpressors of *PhFT4*-*YFP* and *PhFT5*-*YFP* flowered slightly earlier than wild type at 29.1 ± 1.2 and 32.2 ± 3 days with 13.2 ± 1.4 and 14 ± 2.1 rosette leaves, respectively (Fig. 4b,c). Similar results were obtained in independent lines for each genes (Fig. S4). Therefore, like other PEBP genes, moso bamboo TFL-like clade genes negatively regulate floral induction in Arabidopsis, and the moso bamboo FT-like clade gene and MFT-like clade gene promote it.

**Figure 5:**
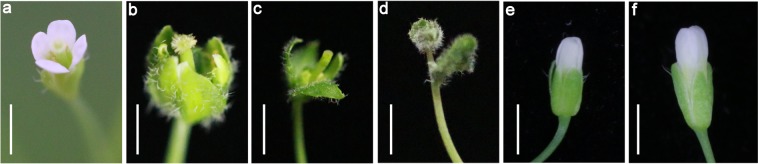
TFL1-like clade genes from moso bamboo regulate floral organ development in Arabidopsis. The floral morphology of Col-0 (**a**) and the overexpressors of PhFT1 (**b**), PhFT2 (**c**), PhFT3 (**d**), PhFT4 (**e**) and PhFT5 (**f**). Scale bar = 1 mm.

In Arabidopsis, TFL-like genes regulate flower organ development. AtTFL1 overexpressor exhibits abnormal flower morphology^44,45^. The overexpressors of *PhFT1*-*YFP*, *PhFT2*-*YFP* and *PhFT3*-*YFP* were also defective in normal flower organ development. *PhFT1*-*YFP*, *PhFT2*-*YFP* and *PhFT3*-*YFP* overexpression lines have leaf-like sepals with lacking petals, although they form normal stamen and pistil (Fig. 5). These results suggest that TFL-like clade genes in moso bamboo also play a function in regulating flower organ development.

### The PhFT5 is induced by ABA during arabidopsis seed germination

**Figure 6:**
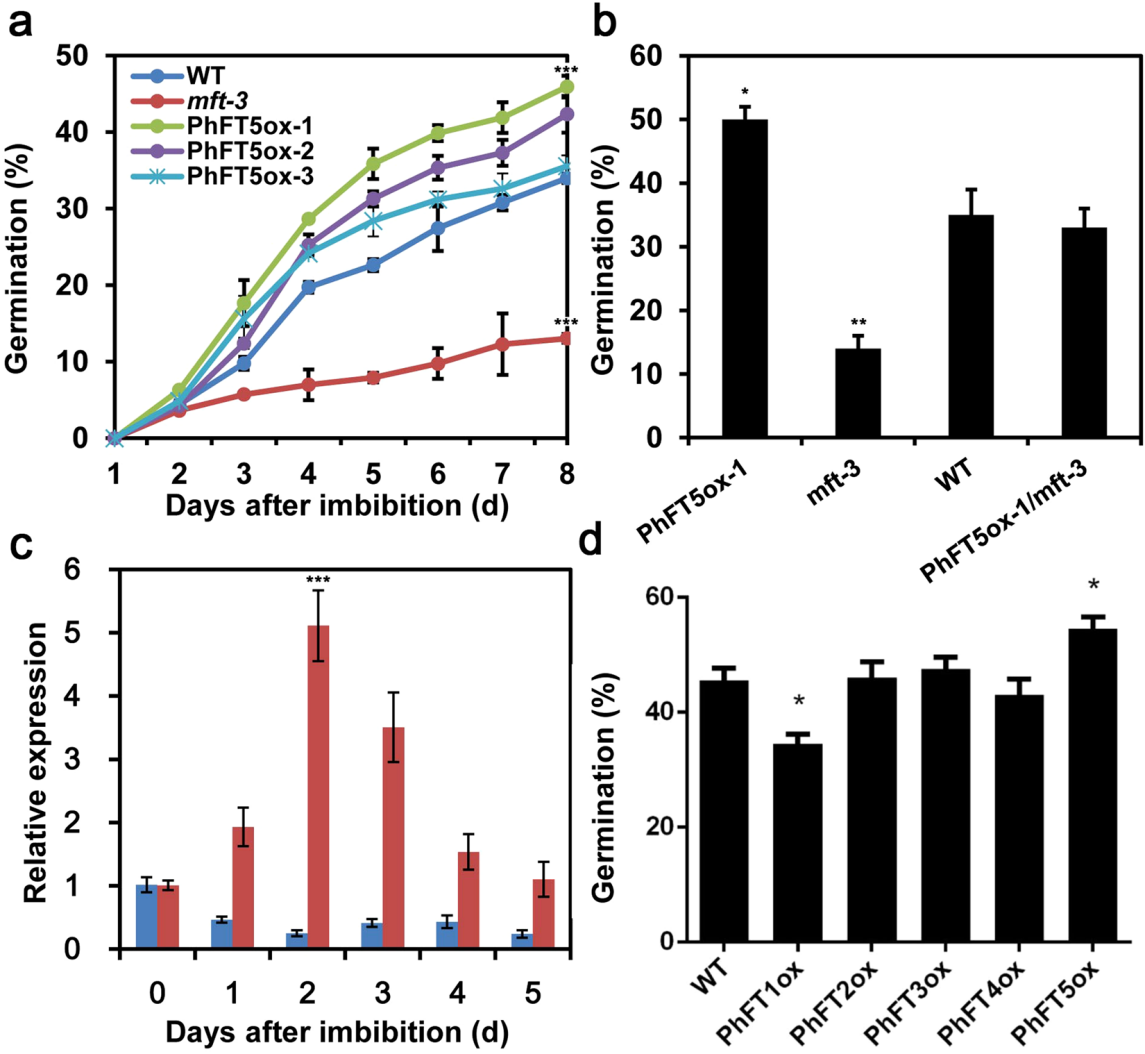
*PhPEBPs* regulate seed germination in Arabidopsis. (**a**) Time course of germination rate for wild type Arabidopsis and 3 representative *PhFT5* overexpression lines in the presence of 10 μM ABA. Bars represent SD from three independent biological repeats. (**b**) Overexpression of *PhFT5* rescues low seed germination rate of *mft*-*3* mutant in the presence of 10 μM ABA in Arabidopsis. Bars represent SD from three independent biological repeats. (**c**) *PhFT5* expression dynamics in moso bamboo seed in the absence (blue) or presence (red) of 100 μM ABA. Bars represent SD from three independent biological repeats (n = 50). (**d**) Comparison of seed germination among wild type Arabidopsis and the overexpressors of *PhFT5* and other *PhPEBPs* in the presence of 10 μM ABA. Bars represent SD from three independent biological repeats (n = 50). Seeds sowed on ½ MS (0.8% agar) medium with ABA kept in the dark at 4 °C for 3 days for stratification, and then transferred to 16 h light/8 h dark photoperiod at 21 °C for 5 days. *, ** and *** denote statistically significant differences determined by Student’s t test (P < 0.05), (P<0.01) and (P < 0.001), respectively.

Abscisic acid (ABA) negatively regulates seed maturation and germination in many species. MFT is known to be up-regulated upon ABA treatment and negatively regulate seed germination through regulating ABA signaling in Arabidopsis^26^. Therefore, we tested the germination rate of transgenic Arabidopsis expressing *PhFT5*-*YFP* in Col-0 or ABA-hypersensitive *mother of ft and tfl1*-*3 FT* (*mft*-*3*) loss-of-function mutant. In the absence of ABA, the germination rate of tested seeds were close to 100% (Fig. S5). However, in the presence of 10 mM ABA, *PhFT5*-*YFP* overexpressors exhibited a higher germination rate compared to Col-0 (Fig. 6a). In addition, the expression of *PhFT5*-*YFP* restored the phenotype of *mft*-*3* (Figs 6b and S6). Regardless of the background genotypes, the germination rate of *PhFT5*-*YFP* overexpressors correlated with the expression level of *PhFT5*-*YFP* (Figs 6b and S6). To further determine the possible role of PhFT5 in the regulation of seed germination in moso bamboo, we examined the expression of PhFT5 during seed germination in the presence or absence of ABA in moso bamboo. As shown in Fig. 6c, the expression of *PhFT5* was slightly reduced after imbibition in the absence of exogenous ABA, where 40.3% seed germination was induced (Fig. S7). On the other hand, it was up-regulated and peaked at 2 days after imbibition in the presence of exogenous ABA (Fig. 6c), where seed germination was impaired (Fig. S7). This results indicated that PhFT5 plays a conserved function in moso bamboo similar to MFT in Arabidopsis^26^.

The expression pattern of other moso bamboo PEBP family genes was further tested in moso bamboo. Similar to the expression of *PhFT5*, transcript levels of *PhFT1*, *2* and *3* are higher in moso bamboo seeds than in leaves (Fig. S8). However, different from *PhFT5*, *PhFT1*, *2* and *3* are expressed stably irrespective of ABA condition (Fig. S9). Interestingly, Arabidopsis PhFT1 overexpressor exhibited significantly low germination rate compared to wild type Col-0 in the presence of ABA (Figs 6d and S10), whereas the overexpressors of other PEBP family genes except for PhFT5 showed wild type response (Fig. 6d). Notably, PhFT1 overexpressor did not exhibit obvious phenotype in the absence of ABA (Fig. S5), suggesting the potential of PhFT1 as positive regulator of ABA mediated inhibition of seed germination.

## Discussion

### The function of PEBP genes in moso baboo is conserved

In this study, 5 PEBP family genes from moso bamboo database have been characterized and named as as *PhFT1*-*5* (Table S1). The number of PEBP family genes in moso bamboo is less than rice (19)^9^, maize (25)^46^ and poplar (9)^47^, and similar as Arabidopsis (6)^15,21^. The numbers of PEBP family members are different in various species. But, generally, the numbers of PEBP family members in monocots are 3–4 times higher than that in dicots^48^. For example, there are 19 members of PEBP family members in rice^9^, although Arabidopsis has only 6 PEBP family members^23^. Our blast search identified only 5 PEBP genes from moso bamboo database, regardless of the fact that both moso bamboo and rice belong to the monocotyledonous grass family and the genome size of moso bamboo is much larger than that of rice (Fig. 1). The reason why moso bamboo has much lower number of PEBP genes is still not clear. Moso bamboo might have eliminated non-functional PEBP family members to develop sophisticated system during its evolution. Similar situation is also found in other species. For example, the biological function of RCN3, one of rice TFL-like genes, has not been detected in rice^32^. Thus, moso bamboo might have eliminated non-functional PEBP family members to develop sophisticated system during its evolution.

The most of reported PEBP family members had conserved amino acid sequence^49^. Phylogenetic analysis identified *PhFT1*, *PhFT2* and *PhFT3* as *TFL1*-*like* gene, *PhFT4* as *FT*-*like* gene and *PhFT5* as *MFT*-*like* gene (Figs 1 and 2). The PEBP family member from moso bamboo and other species exhibited high sequence homology (Figs 1, 2 and S1). Importantly, Tyr^85^ in AtFT and His^88^ in AtTFL1^28^ that distinguish FT-like from TFL-like are conserved (Fig. 2). These results suggest that *FT*-*like* gene and *TFL1*-*like* gene obtained share conserved protein sequences in moso bamboo. FT and TFL1 homologs play flowering induction and repression function in angiosperms, respectively^50^, such as in rice^31,32,51^, poplar^52–54^, soybean^55–58^, pea^59–61^, grapevine^10,62^, kiwifruit^63^, rose^64,65^ and so on. Consistently, PhFT4 promotes flowering in Arabidopsis, but PhFT1, PhFT2 and PhFT3 inhibit it (Fig. 4b,c). In addition, PhTFL1-like genes also regulate floral organ development in Arabidopsis that is consistent with TFL1 homologs in Arabidopsis^66^, Lombardy poplar^47^, Gentiana^67^, *Bambusa oldhamii*^43^, cucumber^68^, *Chrysanthemum morifolium*^69^, but PhFT4 does not (Fig. 5). Thus, these amino acid residues are also determinant for FT-like and TFL-like functions in moso bamboo. The MFT-like PhFT5 gene also has activity to promote flowering in a heterologous system, albeit weakly (Fig. 4b,c), and promote seed germination in Arabidopsis (Fig. 6a,b) that is similar with AtMFT’s function in Arabidopsis^26,70^. Thus, it is very likely that the regulation of flowering and seeds germination through PEBP family genes in moso bamboo is conserved.

It has been proposed that FT-like can form a protein complex with FD that is a transcription factor, while TFL1-like can interact with 14-3-3 protein, in SAM to promote and inhibit flowering, respectively^23,71,72^. We found three FD homologues and seven 14-3-3 homologues in moso bamboo (Tables S4 and S5). Some of these FD and 14-3-3 like proteins contain SAP motif (R/KXX-pS/TXP) or SAP-like motif (RXXSTQF) (Figs S11 and S12), which are necessary for their physical interaction^71–73^. Actually, key amino acid residues, which comprise the interface for their interaction, are highly conserved (Figs S11 and S12). In addition, SAP motif and 14-3-3 recognition motifs are highly conserved in FT-like and TFL1-like sequences from moso bamboo (Fig. 2). Therefore, we speculate that PhFT4, and moso bamboo TFL1-like genes also form complex with these proteins to regulate flowering, although this regulatory mechanism should be experimentally examined in moso bamboo in the future. Although the molecular mechanisms how MFT-like gene mediate seed germination are currently unknown, PhFT5 may act as co-regulator that modify the transcription factor activity since lack DNA-binding domain as is the case with FT-like and TFL1-like genes since AtMFT regulates the transcription of *ABA INSENSITIVE 5*, *EARLY METHIONINE*-*LABELLED 6* and *RESPONSIVE TO DESICCATION 29A* to induce seed germination^26^. Thus, it is a possibility that the molecular mechanism of PEBP family genes on regulation flowering and seed germination are conserved.

Since the vegetative phase can last several decades and data from direct studies of bamboos are limited, the regulatory events leading to phase transition and induction of flowering is largely unknown. Previous reports have speculated that endogenous factor such as circadian clock has an important effect on synchronizing flowering in bamboo^74^. In addition, phytohormones also regulate flowering time^53^. It has been discovered by *in vitro* tissue culture that cytokinins and auxin play positive and negative role to induce flowering, respectively^75–78^. Besides, salicylic acid and gibberellin, are also slightly effective to induce flower formation^79^. On the other hand, exogenous factors such as temperature, severe stress, drought and nitrogen concentration affect bamboo flowering^80–82^. Whether these endogenous and exogenous signals regulate PEBP family gene expression in moso bamboo is currently unknown. However, *cis*-elements associated with some of these signals are found on the promoter region of PEBP family genes. For example, the PhFT4 promoter region contains *cis*-elements associated with drought response and a putative binding site of FLC, a critical transcription factor regulating vernalization (Table S3)^83^. The promoter regions of TFL-like genes also contains many putative elements related to responses to phytohormone, temperature stress and drought (Table S3), implying that endogenous and exogenous factors may coordinate the transcription of FT-like and TFL1-like genes to induce moso bamboo flowering. Indeed, these expression patterns are robustly changed during transition between vegetative and reproductive phase (Fig. S8).

### PhFT1 plays role in seed germination

Moso bamboo *TFL1*-*like* genes were shown conserved function on flowering repression (Fig. 4), however, it is surprisingly that PhFT1 overexpressor exhibited significantly reduced seed germination rate (Fig. 6d). This is a first discovery indicating the possible involvement of TFL1-like gene in the regulation of seed germination. It should be noted here that PhFT1 and PhFT5 oppositely regulate seed germination (Fig. 6d). This is reminiscent of the relation between function of FT-like genes and TFL1-like genes, in which they compete each other to form complex with FD^72^. However, only PhFT1 among PhTFL1-like genes affects seed germination (Fig. 6d), although all PhTFL1-like genes similarly affect flowering time (Fig. 4b,c), suggesting the different action mode of PhFT1 between the regulation of flowering and germination. Whether Arabidopsis TFL1-like genes also regulate seed germination and what the binding partners of TFL1-like proteins to regulate germination are need to be addressed in the future.

Higher expression of PhTFL1-like genes and PhFT5 in seeds compared to leaf tissue also partially represents the functions of PhFT1 and PhFT5 in the regulation of seed germination (Fig. S8). In Arabidopsis, AtMFT plays a role as desensitizer in the negative feedback loop of ABA-inhibited seed germination, because positively acting *AtMFT* expression is induced by the exogenously applied ABA^26^. *PhFT5* is induced by ABA application in moso bamboo seed (Fig. 6c) and PhFT5 promoter contains ABA responsive *cis*-element (Table S3), suggesting that PhFT5 also constitutes a feedback loop that modifies endogenous ABA sensitivity to regulate embryo growth in moso bamboo. Different from PhFT5, PhFT1 expression is not affected by ABA (Fig. S9). However, the promoter of PhFT1 as well as that of PhFT5 contains *cis*-elements responsive to other hormone, circadian clock and environmental factors such as light and temperature (Table S3). Thus, PhFT1 and PhFT5 may negatively and positively fine-tune the capability of moso bamboo seed germination in the fluctuating environment in nature.

This study provides clues to understand the function of five PEBP family genes from moso bamboo in the regulation of flowering and seed germination. The findings also highlight the possible importance of PEBP family genes in the reproduction, thereby in the maintenance of moso bamboo forest. However, further studies are required to characterize these PEBP family genes by genetic approaches to understand their roles in flowering and germination in moso bamboo.

## Methods

### Sequence alignment and phylogenetic analysis

Full length amino acid sequences of Arabidopsis and rice PEBP family members were used as queries squences to blast against moso bamboo genome (BambooGDB, http://server.ncgr.ac.cn/bamboo/blast.php). The resulting protein sequences with expectation values ≤10^−10^ were used as new queries in a second round of blast search with similar search parameters to ensure detection of all orthologs. After removing redundant sequences, the presence of a PEBP domain was confirmed using SMART (http://smart.embl-heidelberg.de/) before subsequent analysis. The phylogenetic tree was constructed using IQ-TREE 1.6.9^84^ with the JTT + G4 model, the best-fit model as determined by ModelFinder^85^. Genes used in sequence alignment and phylogenetic analysis listed in Supplemental Table 6.

### Plant materials and growth conditions

For gene expression analysis, moso bamboo flowering tissues with different developmental stages were collected from NanPing City (117°58′45″E~118°57′11″E; 26°38′54″N~27°20′26″N), Fujian Province, China in July, 2017 (Fig. S2). *Arabidopsis thaliana* Col-0 were used throughout this study to produce stable transgenic lines. The T-DNA mutant line *mft*-*3* (SALK_024298) was ordered from Arabidopsis Biological Resource Center (http://www.arabidopsis.org). *35S::PhFT5*-*YFP#1* and *35S::PhFT5*-*YFP#3* were introduced into *mft*-*3* by crossing to generate *PhFT5ox*-*1*/*mft*-*3* and *PhFT5ox*-*3*/*mft*-*3*. F2 seeds were sterilized and grown on 1/2 MS (0.8% agar) medium containing 1% (v/v) Basta for 2 weeks. Resistant plants were transferred to soil and the homozygote line were established by PCR-based genotyping with specific primers (Table S2). Homozygous F3 seeds were used for this study. Arabidopsis plants were grown at 21 °C in a long day photoperiod (16 h of light/8 h of dark).

### Seed germination assay

For Arabidopsis, after-ripened seeds were sterilized with 75% (v/v) alcohol for 10 min, and washed once with absolute ethanol for 1 min, then dried in concentrator plus (Eppendorf). For moso bamboo, after-ripened moso bamboo seeds were sterilized with chlorine gas in a vacuum container for 5 hours. After sterilization, at least 50 Arabidopsis or moso bamboo seeds were sowed on 1/2MS (0.8% agar) medium with or without ABA (Sigma-Aldrich) and kept in the dark at 4 °C for 3 days for stratification. Seeds were then transferred to 16 h light/8 h dark photoperiod and 21 °C to examine seed germination. Germination was counted when the radicle emerges.

### RNA extraction and RT-qPCR assay

Total RNA was extracted with TIANGEN RNAprep Pure Plant Kit (DP441) according to the manufacturer’s instructions. 1 μg of total RNA was used for reverse transcription with PrimeScript™ RT reagent Kit (TaKaRa, RR047A). qRT-PCR was performed with GoTaq® qPCR Master Mix (Promega) on a QuantStudio™ 6 Flex Real-Time PCR System. A 40-cycle two-step amplification protocol was used for all measurements. The qPCR signals were normalized to that of the reference gene *PhUBQ* using the ΔCT method. All experiments incorporated three technical replicates and biological replicates. The primer sequences are listed in the supplementary material (Table S2).

### *PhPEBP*s gene cloning and vectors construction

The full-length coding sequences of PhFT1, PhFT2, PhFT3, PhFT4 and PhFT5 were amplified by PCR from cDNA of the moso bamboo seedling with gene specific primers. The amplified PCR products were cloned into pDONR207 vector (Invitrogen) by BP reaction of the Gateway technology (Invitrogen), and then transferred into pEarlyGate 101 binary vector (Invitrogen) by LR reaction (Invitrogen). Primersequences used for plasmid construction are given in Table S2. The obtained binary vectors were transformed into Arabidopsis by Agrobacterium-mediated flower dip method, and resultant transgenic Arabidopsis were screened with 1% (v/v) basta on 1/2 MS (0.8% agar) medium.

### Statistical analysis

Statistically significant differences were computed based on Student’s t-tests.

## Supplementary information

Supplementary information
