# Supplementary material for: Identification and Characterization of the PEBP Family Genes in Moso Bamboo (Phyllostachys heterocycla)
Source: Sci Rep. 2019 Oct 18;9:14998. doi: 10.1038/s41598-019-51278-7 (PMC6802209; doi:10.1038/s41598-019-51278-7)
Supplement: Supplementary file 1 — Supplementary information [file 41598_2019_51278_MOESM1_ESM.docx]

**Supplementary information:**

# Identification and Characterization of the PEBP Family Genes in Moso Bamboo (*Phyllostachys heterocycla*)

# Zhaohe Yang^1,2^, Lei Chen^1,2^, Markus V. Kohnen^2^, Bei Xiong^2,3^, Xi Zhen^2,3^, Jiakai Liao^2,3^, Yoshito Oka^2^, Qiang Zhu^2^, Lianfeng Gu^2^, Chentao Lin^2,4,^* and Bobin Liu^1,2,^*

1 College of Forestry, Fujian Agriculture and Forestry University, Fuzhou 350002, Fujian, China

2 Basic Forestry and Proteomics Research Center, Fujian Agriculture and Forestry University, Fuzhou 350002, Fujian, China

3 College of Life Science, Fujian Agriculture and Forestry University, Fuzhou 350002, Fujian, China

4 Department of Molecular, Cell & Developmental Biology, University of California, Los Angeles, CA90095, USA

* Correspondence and requests for materials should be addressed to Chentao Lin (email: clin@mcdb.ucla.edu) and Bobin Liu (email: liubobin@fafu.edu.cn)


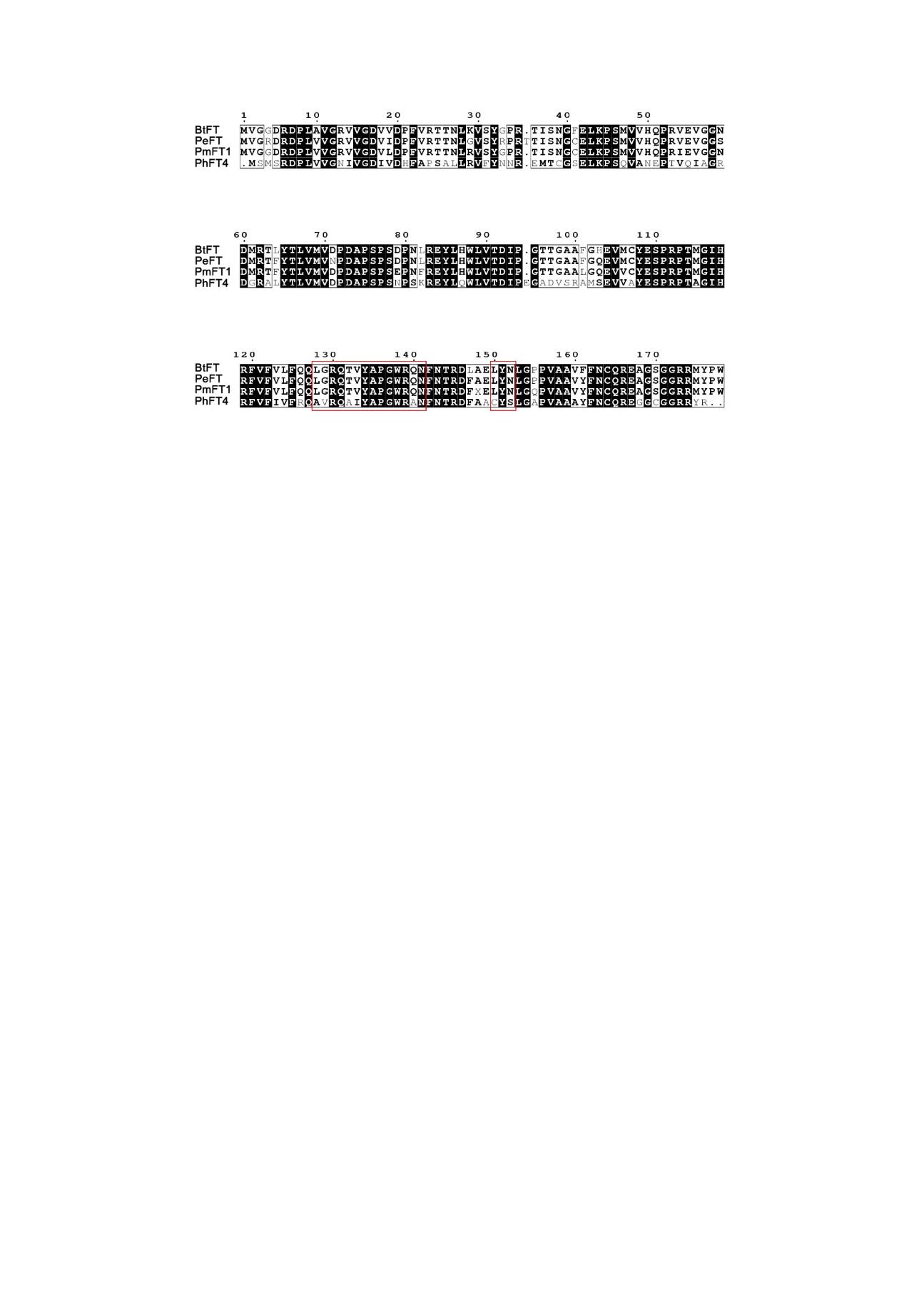


**Supplementary Figure 1.** Sequence comparison of FT-like proteins among bamboo species. Amino acid alignment of FT-like proteins from *Phyllostachys heterocycla* (Ph)[^1^](#_ENREF_1), *Bambusa tulda* (Bt)[^2^](#_ENREF_2), *Phyllostachys edulis* (Pe) and *Phyllostachys meyeri* (Pm)[^3^](#_ENREF_3) is shown. Red boxes represent segment B and triplet module L/IYN, respectively.


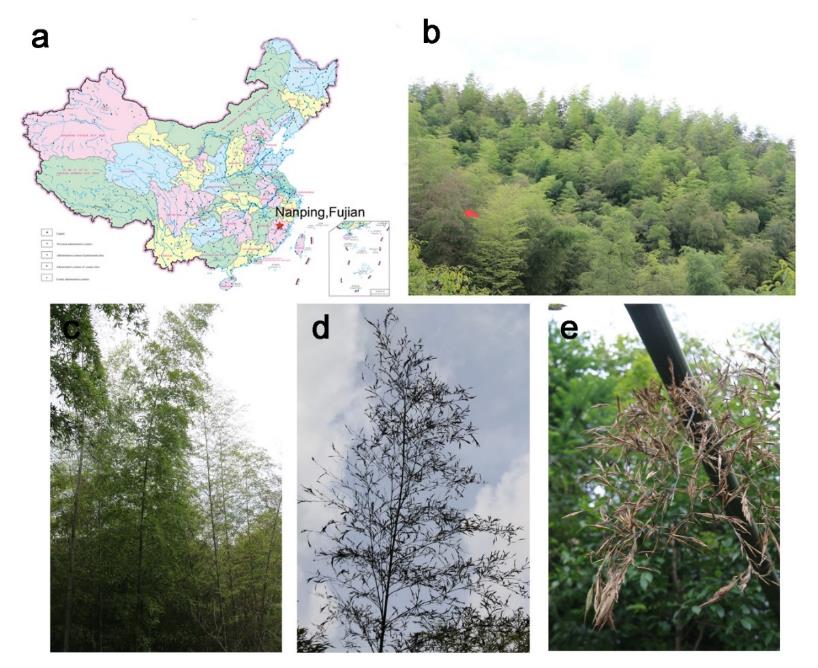


**Supplementary Figure 2.** The location for flowering moso bamboo sampling. (**a**) The red star indicates the location of Nanping city, where flowering moso bamboo was harvested. (**b**) Picture showing the bamboo forestry. The red arrows indicate the flowered bamboo. (**c**-**e**) The development situation of moso bamboo. c, leaf before flowering. d, flowering tissue. e, seed.

**
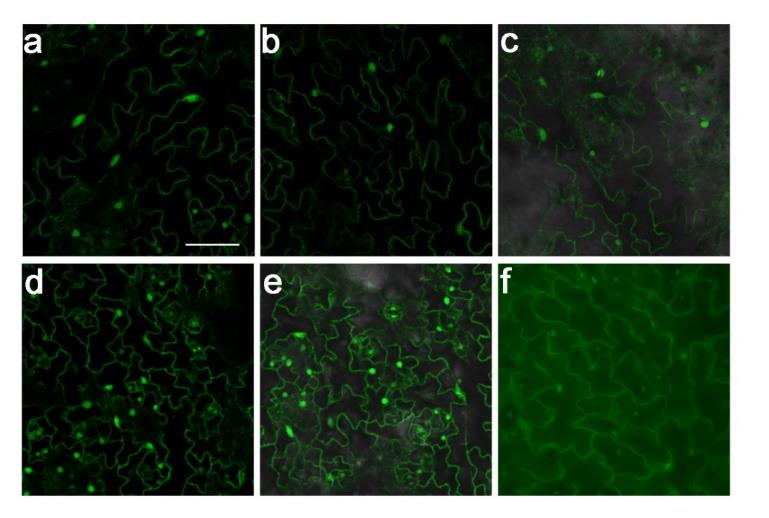
**

**Supplementary Figure 3.** Subcellular localization of PhPEBPs proteins in Arabidopsis lower epidermal cells. The confocal microscopic images show that nuclear and cytoplasm localization of PhFT1-YFP (**a**), PhFT2-YFP (**b**), PhFT3-YFP (**c**), PhFT4-YFP (**d**), PhFT5-YFP (**e**) and YFP (**f**). Scale bar = 20μm.


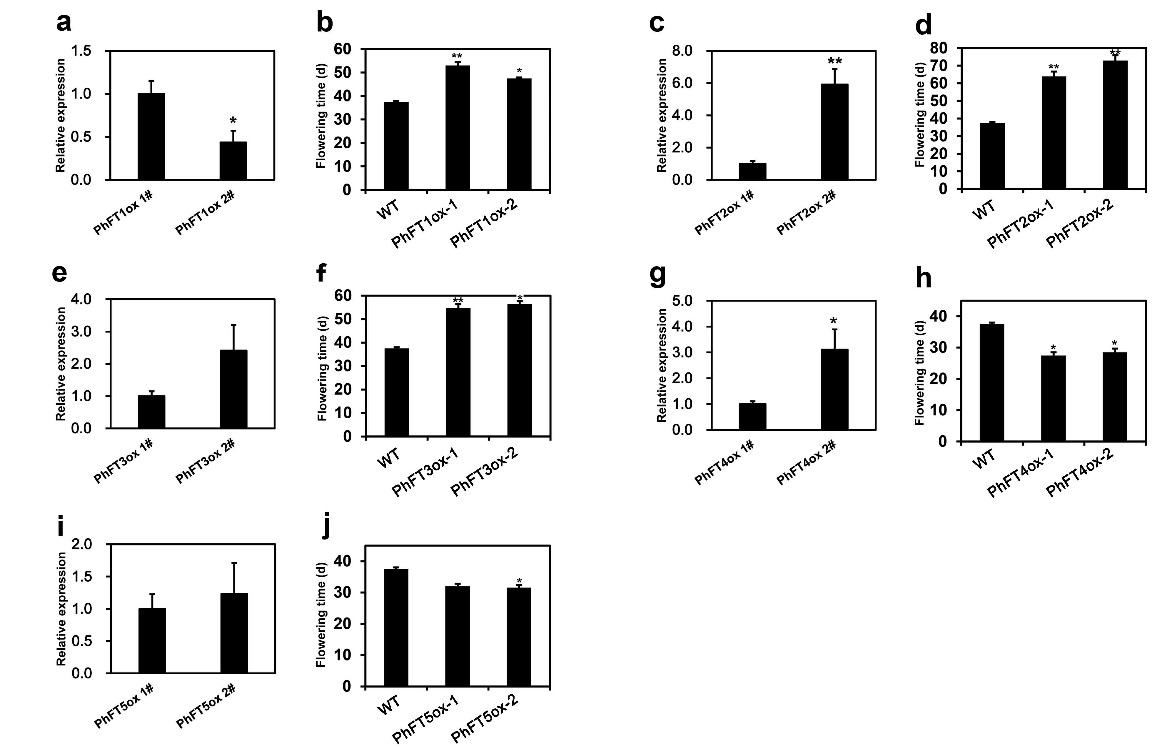


**Supplementary Figure 4.** Overexpression of PhPEBPs alter flowering time in Arabidopsis. (**a**, **c**, **e**, **g** and **i**) qPCR analysis showing the expression level of *PhFT1* (**b**), *PhFT2* (**d**), *PhFT3* (**f**), *PhFT4* (**h**) and *PhFT5* (**i**) in seedlings of two representative transgenic lines. The expression of the Arabidopsis *UBQ* gene was used as an internal control. (**b, d**, **f**, **h** and **j**) Days to flower of *PhFT1* (**b**), *PhFT2* (**d**), *PhFT3* (**f**), *PhFT4* (**h**) and *PhFT5* (**j**) overexpressing plants grown under long day condition for 38-day-old. * and ** denote significant differences (P<0.05) and (P < 0.01), respectively.


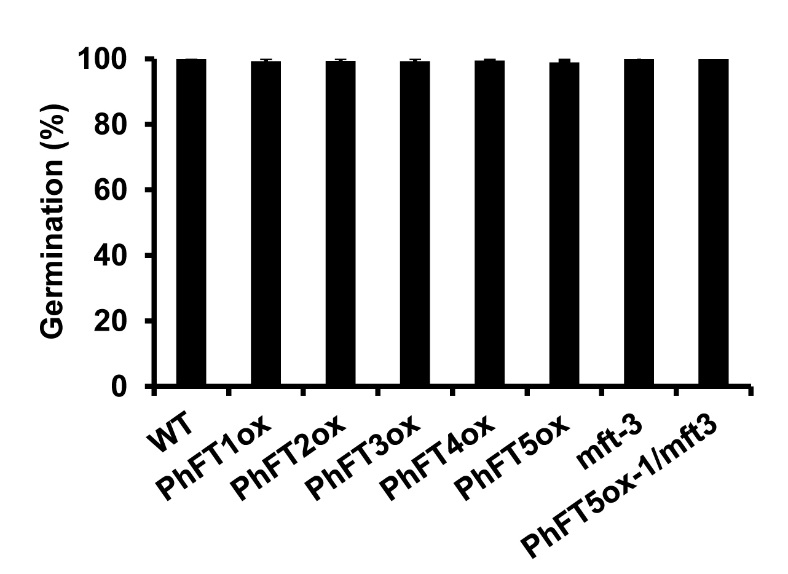


**Supplementary Figure 5.** Seeds germination ratio analysis of *PhPEBPs* overexpression in Arabidopsis. All seeds grown on ½ MS media without ABA were stratified in darkness at 4°C for 3 days and then transferred to 16 h light / 8 h dark condition at 21°C for 5 days. Bars represent SD from 3 independently biological repeat, n=50 for every repeat.


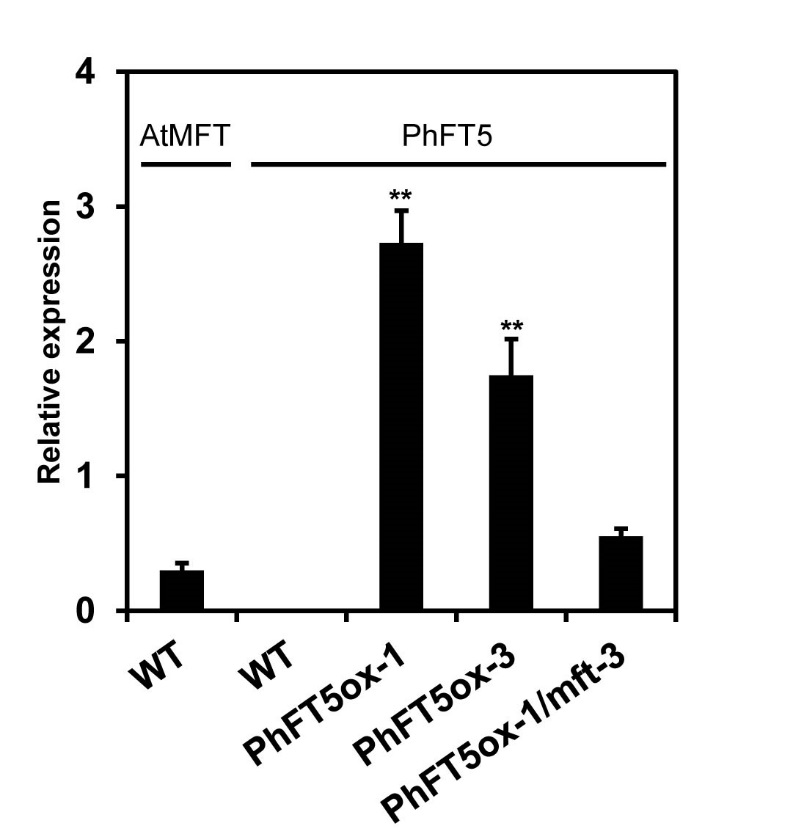


**Supplementary Figure 6.** Expression level of *PhFT5*in *PhFT5ox* lines and *PhFT5ox*/*mft-3.* qPCR analysis showing the transcriptional level of *PhFT5* in two representative *PhFT5ox* lines and *PhFT5ox*/*mft-3.* The qPCR signal were normalized against Arabidopsis *UBQ*, the error bars represent the SD from three biological repeats. ** denote significant differences (P < 0.01).


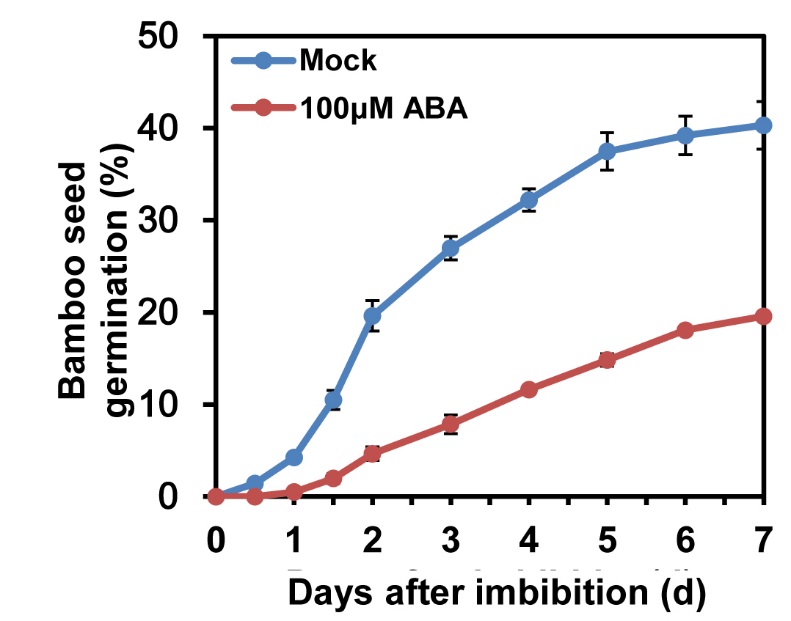


**Supplementary Figure 7.** Germination phenotype of moso bamboo seed under absent or present 100 μM ABA treatment. Bars represent ± SD from three independent biological repeats. n=50 for each repeat.


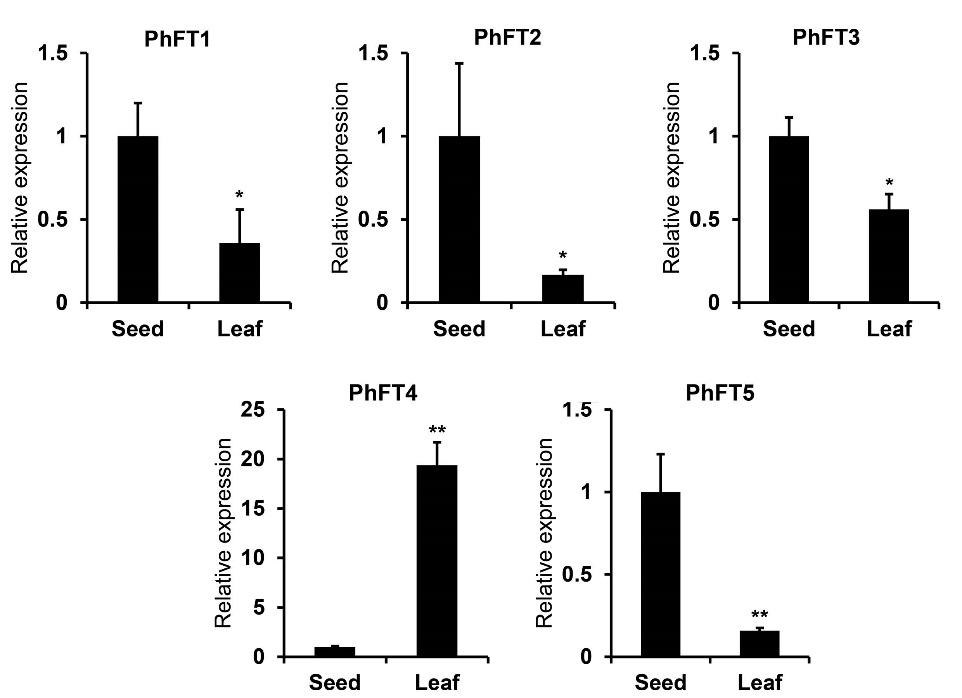


**Supplementary Figure 8.** Expression analysis of *PhPEBPs* in moso bamboo. Each *PhPEBPs* expression pattern in seed and leaf before flowering of moso bamboo. qRT-PCR results show *PhFT1*, *PhFT2*, *PhFT3* and *PhFT5* highly expressed in moso bamboo seed, while *PhFT4* are highly expressed in leafs. The qPCR signal of every *PhPEBP’* expression was normalized to *PhUBQ*, the bars represent mean ± SD from three biological repeats. * and ** denote significant differences (P<0.05) and (P < 0.01), respectively.


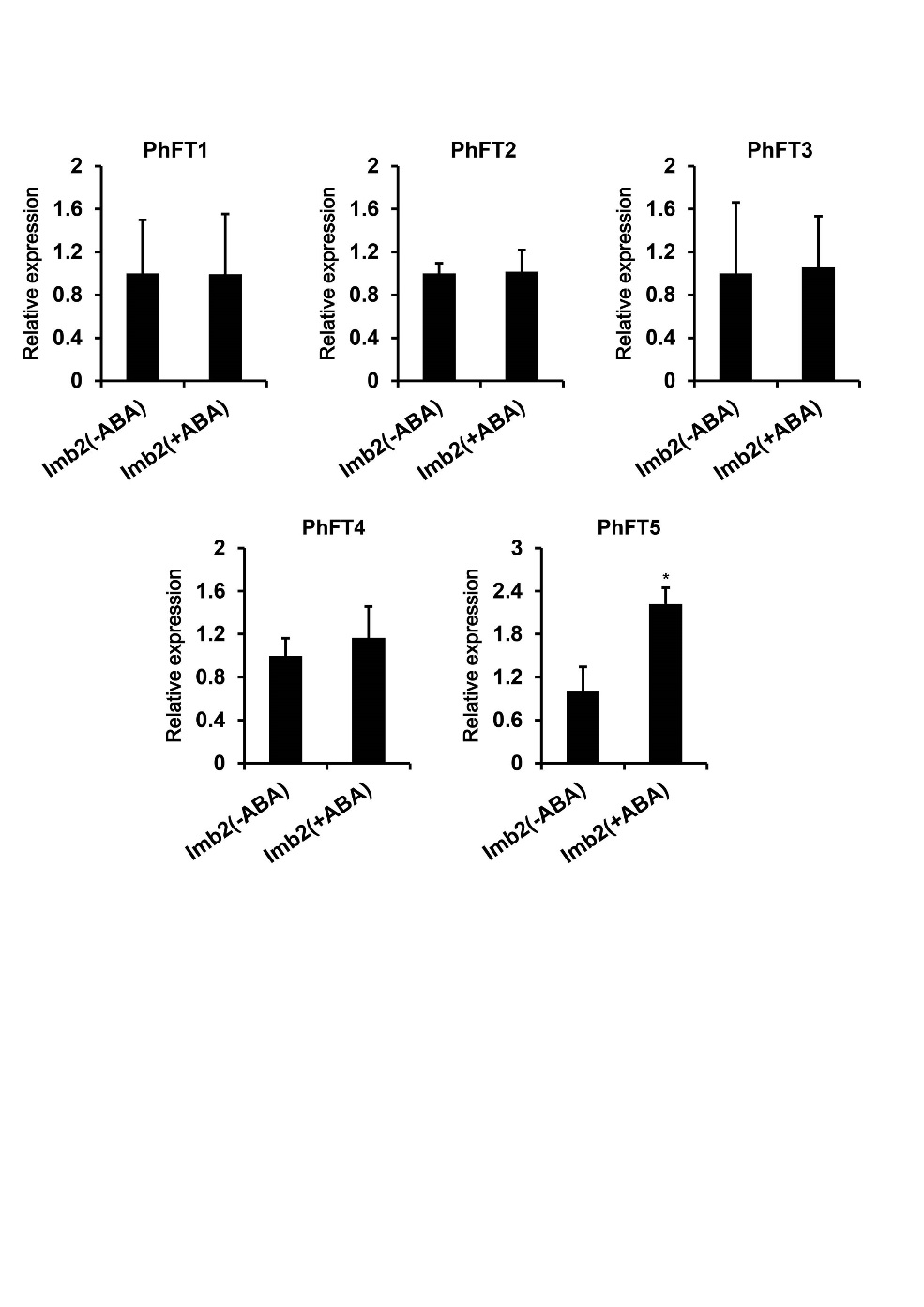


**Supplementary Figure 9.** *PhPEBPs* expression dynamic in response to ABA. qRT-PCR results show that *PhFT5* expression is upregulated in response to ABA. The qPCT signals normalized to *PhUBQ*, the bars represent mean ± SD from three biological repeats. All seeds were collected 2 days after imbibition with or without 10µM ABA treatment. * denote significant differences (P<0.05).


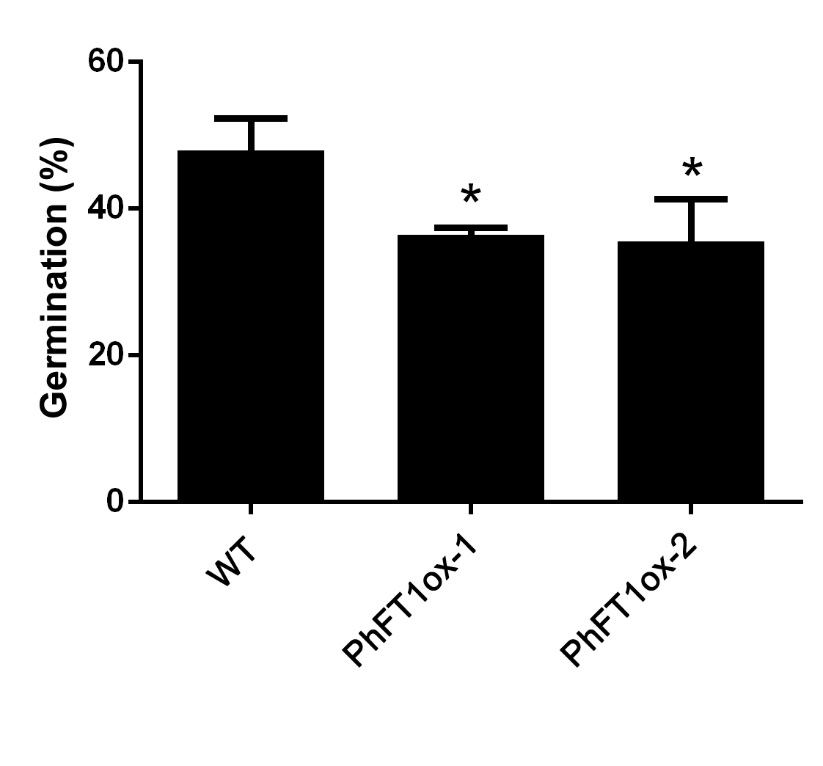


**Supplementary Figure 10.** Germination rate for wild type Arabidopsis and 2 representative *PhFT1* overexpression lines in the presence of 10 μM ABA. Bars represent SD from three independent biological repeats (n=50). Seeds sowed on 1/2MS (0.8% agar) medium with ABA kept in the dark at 4°C for 3 days for stratification, and then transferred to 16 h light / 8 h dark photoperiod at 21°C for 5 days. Asterisk indicates 0.01 < p < 0.05.


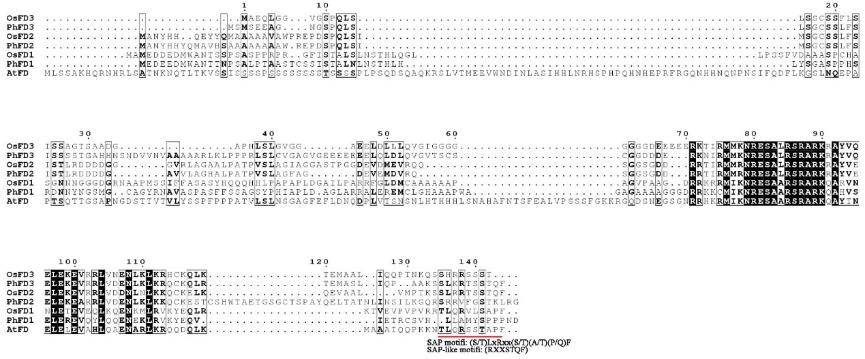


**Supplementary Figure 11.** Full length alignment analysis of FD proteins from *Phyllostachys heterocyclea* (Ph), *A. thaliana* (At), *Oryza sativa* (Os). The SAP-like motif (RXXSTQF) that is conserved in PhFD3 is underlined in red.


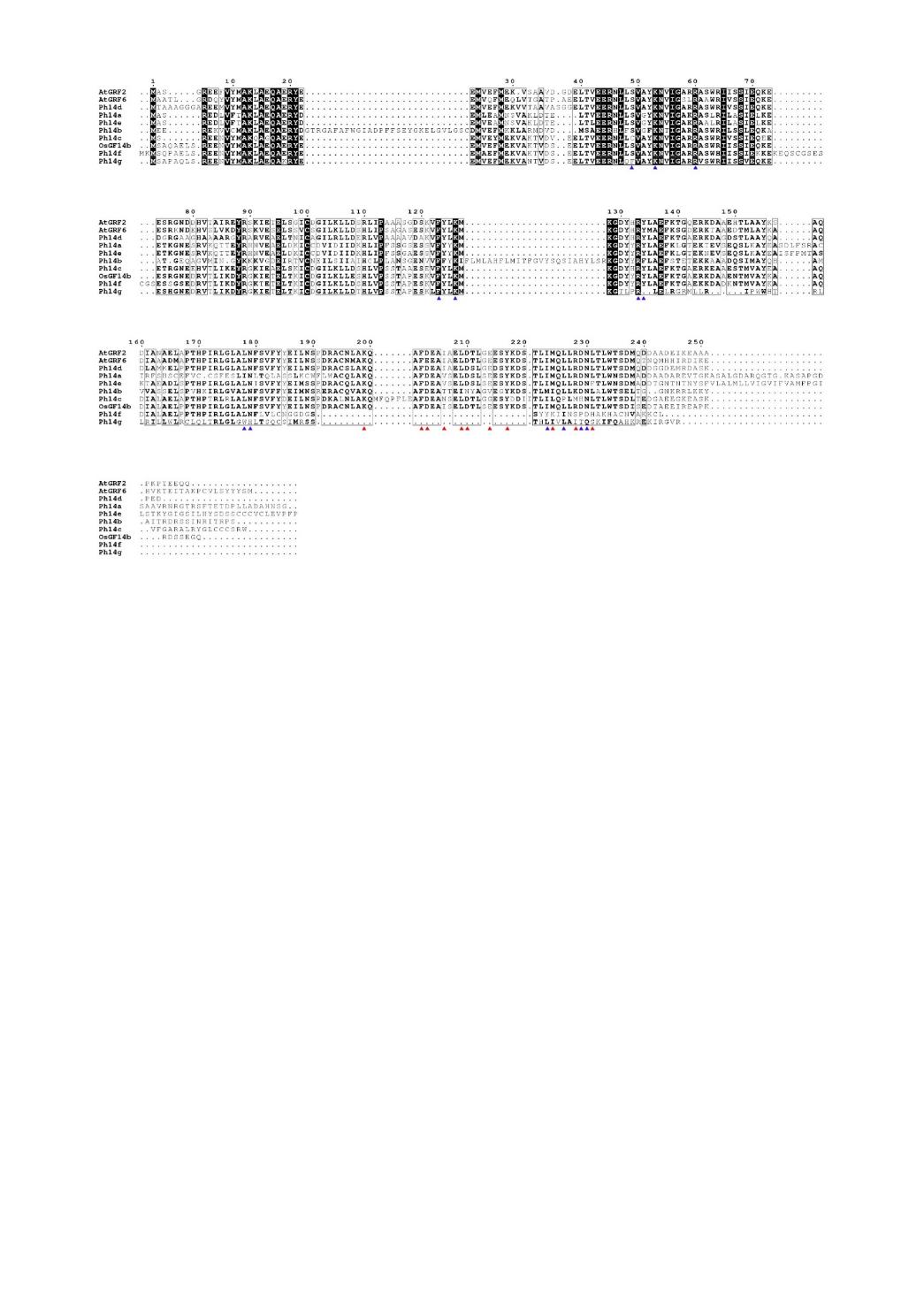


**Supplementary Figure 12.** Full length alignment analysis of 14-3-3 proteins from *Phyllostachys heterocyclea* (Ph), *A. thaliana* (At), *Oryza sativa* (Os). Ph14a, Ph14b, Ph14c, Ph14d, Ph14e, Ph14f and Ph14g, 2 Arabidopsis 14-3-3 proteins, and 1 rice 14-3-3 protein were analyzed. Residues marked by blue or red triangles are the motif interacted with FD. Note that there are conserved FD interaction motif in Ph14a, Ph14b, Ph14c, Ph14d proteins.


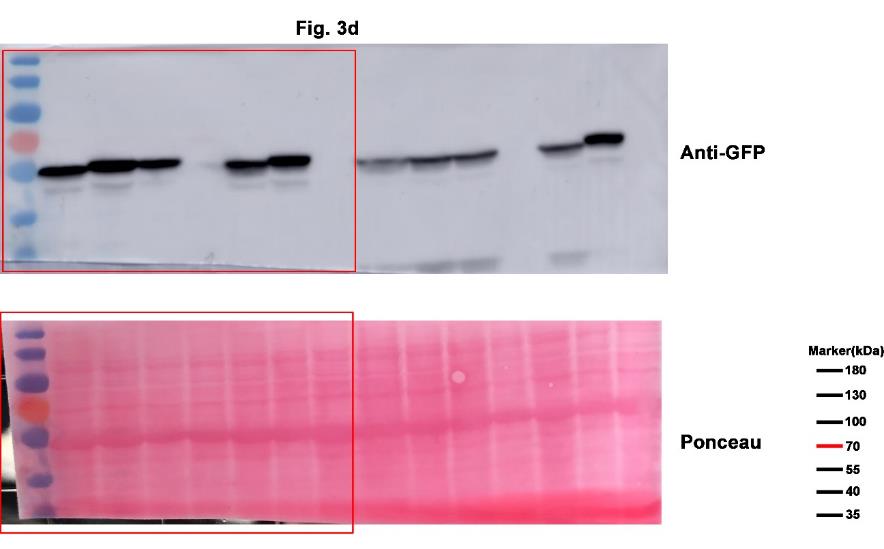


**Supplementary Figure 13.** Full scan data of immunoblot. Red boxes represent the data shown in figure 3d.

**Supplementary Table** **1.** Comparison of the *Ph*FTs and the FT proteins of Arabidopsis and rice.

| Name | BamboGDB | Protein size (aa) | Full-length amino acid identity (%) | | | | | |
| --- | --- | --- | --- | --- | --- | --- | --- | --- |
|  |  |  | *At*FT | *At*TFL1 | *At*MFT | *Os*Hd3a | *Os*RCN1 | *Os*MFT |
| *Ph*FT1 | PH01001134G0390 | 173 | 56.82 | 71.51 | 52.57 | 59.44 | 98.27 | 50.00 |
| *Ph*FT2 | PH01003363G0220 | 173 | 55.68 | 70.39 | 52.57 | 58.33 | 96.53 | 50.00 |
| *Ph*FT3 | PH01002570G0010 | 173 | 51.14 | 71.35 | 50.29 | 56.11 | 83.24 | 51.12 |
| *Ph*FT4 | PH01002288G0050 | 176 | 61.58 | 46.41 | 47.19 | 60.00 | 49.15 | 49.15 |
| *Ph*FT5 | PH01007086G0020 | 177 | 44.69 | 50.00 | 66.67 | 51.91 | 51.96 | 88.14 |

**Supplementary Table** **2.** List of primers used in this study.

| **Name** | **primer sequence (from 5′ to 3′)** | **Purpose** |
| --- | --- | --- |
| PhFT1-F | GGGGACAAGTTTGTACAAAAAAGCAGGCTTCATGTCTAGGTCTGTGGAGCCTCTCATT | Preparing PhFTs-YFP binary vectors |
| PhFT1-R | GGGGACCACTTTGTACAAGAAAGCTGGGTCGCGCCTCCTGGCAGCAGTCTC |  |
| PhFT2-F | GGGGACAAGTTTGTACAAAAAAGCAGGCTTCATGTCTAGGTCTGTGGAGCCTCTCATTG |  |
| PhFT2-R | GGGGACCACTTTGTACAAGAAAGCTGGGTCGCGTCTCCTGGCAGCAGTCTC |  |
| PhFT3-F | GGGGACAAGTTTGTACAAAAAAGCAGGCTTCATGTCTAGGGTGCTGGAGCCTCT |  |
| PhFT3-R | GGGGACCACTTTGTACAAGAAAGCTGGGTCGCGGCGGCGTGCGGCGGTCTC |  |
| PhFT4-F | GGGGACAAGTTTGTACAAAAAAGCAGGCTTCATGAGCATGTCGAGGGATCCGC |  |
| PhFT4-R | GGGGACCACTTTGTACAAGAAAGCTGGGTCCCTGTACCTCCGGCCACCGC |  |
| PhFT5-F | GGGGACAAGTTTGTACAAAAAAGCAGGCTTCATGGCAGCCCATGTGGATCCG |  |
| PhFT5-R | GGGGACCACTTTGTACAAGAAAGCTGGGTCGTAGTGGCGGCGGCGGTT |  |
| PhFT1-qF | ACA GAC GCT TCT TTT GGA CG | RT-qPCR |
| PhFT1-qR | ATT CTC CTC AGC AAA ACG GC |  |
| PhFT2-qF | GCG GGT GAT TGG AGA AGT TC |  |
| PhFT2-qR | CCA ATG TGA AGA AGG ACC GC |  |
| PhFT3-qF | GGC CAC GAA TTC TTC CCA TC |  |
| PhFT3-qR | AGC TCA CCA CCT CAT TTC CA |  |
| PhFT4-qF | AAC AAC CGC GAG ATG ACT TG |  |
| PhFT4-qR | CCT CAG ACA TTG CAC GAC TG |  |
| PhFT5-qF | CAT GTG GAT CCG CTT GTG G |  |
| PhFT5-qR | TCC CTC ATG TTT GGC TCA CT |  |
| PhUBQ-F | GGGTCGTCCAGTGTCCTCTATTA |  |
| PhUBQ-R | TCAACCAAACCACTGTACCTCAG |  |
| mft-3LP | TACCTCTTGAGGGATTTGTGC | mft-3 genotyping |
| mft-3RP | TCTGTTGATCCTTTGGTGGTC |  |
| LBb1.3 | ATTTTGCCGATTTCGGAAC |  |

**Supplementary Table** **3.** Promoter analysis of moso bamboo PEBP family genes.

| **Gene** | **Site Name** | **Sequence** | **Position** | **Function** |
| --- | --- | --- | --- | --- |
| ***Ph*FT1** | CGTCA-motif | CGTCA | 1076 | cis-acting regulatory element involved in the MeJA-responsiveness |
|  | CGTCA-motif | CGTCA | 1585 | cis-acting regulatory element involved in the MeJA-responsiveness |
|  | A-box | CCGTCC | 973 | cis-acting regulatory element |
|  | AE-box | AGAAACAA | 924 | part of a module for light response |
|  | GARE-motif | TCTGTTG | 1430 | gibberellin-responsive element |
|  | I-box | TAGATAACC | 999 | part of a light responsive element |
|  | Box 4 | ATTAAT | 641 | part of a conserved DNA module involved in light responsiveness |
|  | TGACG-motif | TGACG | 1076 | cis-acting regulatory element involved in the MeJA-responsiveness |
|  | TGACG-motif | TGACG | 1585 | cis-acting regulatory element involved in the MeJA-responsiveness |
|  | MYC | CATTTG | 342 |  |
|  | MYC | CATTTG | 947 |  |
|  | W box | TTGACC | 281 |  |
|  | TC-rich repeats | ATTCTCTAAC | 1831 | cis-acting element involved in defense and stress responsiveness |
|  | GCN4_motif | TGAGTCA | 646 | cis-regulatory element involved in endosperm expression |
|  | G-Box | CACGTT | 1715 | cis-acting regulatory element involved in light responsiveness |
|  | GATA-motif | GATAGGA | 1196 | part of a light responsive element |
|  | GATA-motif | GATAGGA | 1435 | part of a light responsive element |
|  | ARE | AAACCA | 364 | cis-acting regulatory element essential for the anaerobic induction |
|  | ARE | AAACCA | 574 | cis-acting regulatory element essential for the anaerobic induction |
|  | MBS | CAACTG | 822 | MYB binding site involved in drought-inducibility |
|  | MBS | CAACTG | 1288 | MYB binding site involved in drought-inducibility |
|  | TCA-element | CCATCTTTTT | 286 | cis-acting element involved in salicylic acid responsiveness |
|  | TGA-element | AACGAC | 1731 | auxin-responsive element |
|  | Myb-binding site | CAACAG | 411 |  |
|  | Myb-binding site | CAACAG | 1430 |  |
|  | ABRE | ACGTG | 1715 | cis-acting element involved in the abscisic acid responsiveness |
| ***Ph*FT2** | CGTCA-motif | CGTCA | 654 | cis-acting regulatory element involved in the MeJA-responsiveness |
|  | CGTCA-motif | CGTCA | 1040 | cis-acting regulatory element involved in the MeJA-responsiveness |
|  | CGTCA-motif | CGTCA | 1058 | cis-acting regulatory element involved in the MeJA-responsiveness |
|  | CGTCA-motif | CGTCA | 1664 | cis-acting regulatory element involved in the MeJA-responsiveness |
|  | P-box | CCTTTTG | 1524 | gibberellin-responsive element |
|  | AE-box | AGAAACAA | 1812 | part of a module for light response |
|  | Box 4 | ATTAAT | 1226 | part of a conserved DNA module involved in light responsiveness |
|  | TCCC-motif | TCTCCCT | 1083 | part of a light responsive element |
|  | TGACG-motif | TGACG | 654 | cis-acting regulatory element involved in the MeJA-responsiveness |
|  | TGACG-motif | TGACG | 1040 | cis-acting regulatory element involved in the MeJA-responsiveness |
|  | TGACG-motif | TGACG | 1058 | cis-acting regulatory element involved in the MeJA-responsiveness |
|  | Sp1 | GGGCGG | 1012 | light responsive element |
|  | TC-rich repeats | ATTCTCTAAC | 1804 | cis-acting element involved in defense and stress responsiveness |
|  | G-Box | CACGTG | 987 | cis-acting regulatory element involved in light responsiveness |
|  | GATA-motif | GATAGGA | 192 | part of a light responsive element |
|  | GATA-motif | AAGGATAAGG | 1132 | part of a light responsive element |
|  | O2-site | GATGATGTGG | 573 | cis-acting regulatory element involved in zein metabolism regulation |
|  | G-box | CACGTG | 987 | cis-acting regulatory element involved in light responsiveness |
|  | G-box | CACGTC | 1041 | cis-acting regulatory element involved in light responsiveness |
|  | LTR | CCGAAA | 1179 | cis-acting element involved in low-temperature responsiveness |
|  | MBS | CAACTG | 56 | MYB binding site involved in drought-inducibility |
|  | MBS | CAACTG | 602 | MYB binding site involved in drought-inducibility |
|  | MBS | CAACTG | 814 | MYB binding site involved in drought-inducibility |
|  | MBS | CAACTG | 1799 | MYB binding site involved in drought-inducibility |
|  | TGA-element | AACGAC | 1167 | auxin-responsive element |
|  | TGA-element | AACGAC | 1699 | auxin-responsive element |
|  | ABRE | GACACGTACGT | 655 | cis-acting element involved in the abscisic acid responsiveness |
|  | ABRE | CACGTG | 987 | cis-acting element involved in the abscisic acid responsiveness |
|  | ABRE | ACGTG | 988 | cis-acting element involved in the abscisic acid responsiveness |
|  | ABRE | ACGTG | 1042 | cis-acting element involved in the abscisic acid responsiveness |
| ***Ph*FT3** | ACE | GCGACGTACC | 251 | cis-acting element involved in light responsiveness |
|  | CAT-box | GCCACT | 230 | cis-acting regulatory element related to meristem expression |
|  | CAT-box | GCCACT | 1444 | cis-acting regulatory element related to meristem expression |
|  | P-box | CCTTTTG | 118 | gibberellin-responsive element |
|  | P-box | CCTTTTG | 1574 | gibberellin-responsive element |
|  | I-box | gGATAAGGTG | 188 | part of a light responsive element |
|  | WRE3 | CCACCT | 187 |  |
|  | CCAAT-box | CAACGG | 499 | MYBHv1 binding site |
|  | Sp1 | GGGCGG | 1255 | light responsive element |
|  | TC-rich repeats | GTTTTCTTAC | 1487 | cis-acting element involved in defense and stress responsiveness |
|  | CArG box | CTTTTGG | 426 | Flowering related protein FLC binding site |
|  | Box III | atCATTTTCACt | 1148 | protein binding site |
|  | G-Box | CACGTT | 844 | cis-acting regulatory element involved in light responsiveness |
|  | GATA-motif | GATAGGG | 129 | part of a light responsive element |
|  | GATA-motif | AAGGATAAGG | 190 | part of a light responsive element |
|  | G-box | TACGTG | 960 | cis-acting regulatory element involved in light responsiveness |
|  | G-box | TACGTG | 989 | cis-acting regulatory element involved in light responsiveness |
|  | G-box | TAACACGTAG | 1002 | cis-acting regulatory element involved in light responsiveness |
|  | G-box | TACGTG | 1003 | cis-acting regulatory element involved in light responsiveness |
|  | ARE | AAACCA | 902 | cis-acting regulatory element essential for the anaerobic induction |
|  | ARE | AAACCA | 1562 | cis-acting regulatory element essential for the anaerobic induction |
|  | LTR | CCGAAA | 559 | cis-acting element involved in low-temperature responsiveness |
|  | LTR | CCGAAA | 1389 | cis-acting element involved in low-temperature responsiveness |
|  | TCA-element | CCATCTTTTT | 100 | cis-acting element involved in salicylic acid responsiveness |
|  | TGA-element | AACGAC | 1186 | auxin-responsive element |
|  | TCT-motif | TCTTAC | 804 | part of a light responsive element |
|  | TCT-motif | TCTTAC | 827 | part of a light responsive element |
|  | ABRE | ACGTG | 845 | cis-acting element involved in the abscisic acid responsiveness |
|  | ABRE | ACGTG | 960 | cis-acting element involved in the abscisic acid responsiveness |
|  | ABRE | ACGTG | 989 | cis-acting element involved in the abscisic acid responsiveness |
|  | ABRE | ACGTG | 1004 | cis-acting element involved in the abscisic acid responsiveness |
| ***Ph*FT4** | CGTCA-motif | CGTCA | 1267 | cis-acting regulatory element involved in the MeJA-responsiveness |
|  | CAT-box | GCCACT | 78 | cis-acting regulatory element related to meristem expression |
|  | CAT-box | GCCACT | 1952 | cis-acting regulatory element related to meristem expression |
|  | 3-AF1 binding site | TAAGAGAGGAA | 1294 | light responsive element |
|  | Box 4 | ATTAAT | 989 | part of a conserved DNA module involved in light responsiveness |
|  | HD-Zip 3 | GTAAT(G/C)ATTAC | 200 | protein binding site |
|  | TGACG-motif | TGACG | 1267 | cis-acting regulatory element involved in the MeJA-responsiveness |
|  | CAG-motif | GAAAGGCAGAC | 1869 | part of a light response element |
|  | TC-rich repeats | ATTCTCTAAC | 1466 | cis-acting element involved in defense and stress responsiveness |
|  | ATC-motif | AGTAATCT | 924 | part of a conserved DNA module involved in light responsiveness |
|  | CArG box | CTTTTGG | 1469 | Flowering related protein FLC binding site |
|  | G-Box | CACGTT | 302 | cis-acting regulatory element involved in light responsiveness |
|  | G-Box | CACGTT | 1228 | cis-acting regulatory element involved in light responsiveness |
|  | CARE | CAACTCCC | 1750 |  |
|  | GATA-motif | AAGATAAGATT | 1632 | part of a light responsive element |
|  | G-box | CACGAC | 1660 | cis-acting regulatory element involved in light responsiveness |
|  | GT1-motif | GGTTAAT | 1692 | light responsive element |
|  | GT1-motif | GGTTAA | 1693 | light responsive element |
|  | HD-Zip 1 | CAAT(A/T)ATTG | 321 | element involved in differentiation of the palisade mesophyll cells |
|  | ARE | AAACCA | 896 | cis-acting regulatory element essential for the anaerobic induction |
|  | MBS | CAACTG | 173 | MYB binding site involved in drought-inducibility |
|  | GA-motif | ATAGATAA | 1217 | part of a light responsive element |
|  | TCT-motif | TCTTAC | 1293 | part of a light responsive element |
|  | ABRE | ACGTG | 302 | cis-acting element involved in the abscisic acid responsiveness |
|  | ABRE | ACGTG | 1228 | cis-acting element involved in the abscisic acid responsiveness |
| ***Ph*FT5** | CAT-box | GCCACT | 247 | cis-acting regulatory element related to meristem expression |
|  | CAT-box | GCCACT | 359 | cis-acting regulatory element related to meristem expression |
|  | CAT-box | GCCACT | 723 | cis-acting regulatory element related to meristem expression |
|  | A-box | CCGTCC | 173 | cis-acting regulatory element |
|  | AE-box | AGAAACAA | 581 | part of a module for light response |
|  | GARE-motif | TCTGTTG | 1090 | gibberellin-responsive element |
|  | Box 4 | ATTAAT | 34 | part of a conserved DNA module involved in light responsiveness |
|  | Box 4 | ATTAAT | 1466 | part of a conserved DNA module involved in light responsiveness |
|  | Box 4 | ATTAAT | 1679 | part of a conserved DNA module involved in light responsiveness |
|  | Box 4 | ATTAAT | 1690 | part of a conserved DNA module involved in light responsiveness |
|  | Box 4 | ATTAAT | 1694 | part of a conserved DNA module involved in light responsiveness |
|  | Sp1 | GGGCGG | 159 | light responsive element |
|  | Sp1 | GGGCGG | 1955 | light responsive element |
|  | G-Box | CACGTG | 1732 | cis-acting regulatory element involved in light responsiveness |
|  | GATA-motif | AAGGATAAGG | 1974 | part of a light responsive element |
|  | CARE | CAACTCAC | 1015 |  |
|  | O2-site | GATGATGTGG | 1580 | cis-acting regulatory element involved in zein metabolism regulation |
|  | O2-site | GATGACATGG | 1921 | cis-acting regulatory element involved in zein metabolism regulation |
|  | G-box | CACGTG | 1732 | cis-acting regulatory element involved in light responsiveness |
|  | GT1-motif | GGTTAA | 1340 | light responsive element |
|  | GT1-motif | GGTTAA | 1500 | light responsive element |
|  | GT1-motif | GGTTAAT | 1872 | light responsive element |
|  | GT1-motif | GGTTAA | 1873 | light responsive element |
|  | ARE | AAACCA | 686 | cis-acting regulatory element essential for the anaerobic induction |
|  | TCA-element | CCATCTTTTT | 825 | cis-acting element involved in salicylic acid responsiveness |
|  | TCT-motif | TCTTAC | 432 | part of a light responsive element |
|  | TCT-motif | TCTTAC | 865 | part of a light responsive element |
|  | ABRE | CGCACGTGTC | 1730 | cis-acting element involved in the abscisic acid responsiveness |
|  | ABRE | CACGTG | 1732 | cis-acting element involved in the abscisic acid responsiveness |
|  | ABRE | ACGTG | 1733 | cis-acting element involved in the abscisic acid responsiveness |

**Supplementary Table** **4.** Comparison of the FD proteins of moso bamboo, Arabidopsis and rice.

| Name | BamboGDB | Protein size (aa) | Full-length amino acid identity (%) | | | |
| --- | --- | --- | --- | --- | --- | --- |
|  |  |  | *At*FD | *Os*FD1 | *Os*FD2 | *Os*FD3 |
| *Ph*FD1 | PH01000511G0500 | 187 | 21.68 | 47.55 | 24.61 | 20.31 |
| *Ph*FD2 | PH01001986G0070 | 183 | 10.14 | 13.85 | 64.58 | 31.58 |
| *Ph*FD3 | PH01000642G0890 | 164 | 21.25 | 23.35 | 46.47 | 56.80 |

**Supplementary Table** **5.** Comparison of the 14-3-3 proteins of moso bamboo, Arabidopsis and rice.

| Name | BamboGDB | Protein size (aa) | Full-length amino acid identity (%) | | |
| --- | --- | --- | --- | --- | --- |
|  |  |  | *At*GRF2 | *At*GRF6 | *Os*GF14b |
| Ph14a | PH01002045G0220 | 299 | 47.18 | 43.09 | 47.21 |
| Ph14b | PH01001088G0220 | 314 | 41.07 | 36.39 | 40.56 |
| Ph14c | PH01000008G2370 | 274 | 68.48 | 60.99 | 73.93 |
| Ph14d | PH01001561G0010 | 261 | 75.56 | 63.54 | 70.57 |
| Ph14e | PH01000037G1270 | 302 | 51.15 | 48.38 | 52.10 |
| Ph14f | PH01000146G1150 | 231 | 52.98 | 45.86 | 65.14 |
| Ph14g | PH01007640G0020 | 212 | 41.06 | 34.55 | 50.00 |

**Supplementary Table** **6.** Information of genes used in sequence alignment and phylogenetic analysis.

| **Gene name** | **Species** | **Gene ID in GenBank** | **Reference** |
| --- | --- | --- | --- |
| *At*FT | *Arabidopsis* | NP_176726.1 | [^4^](#_ENREF_4) |
| *At*TSF | *Arabidopsis* | NP_193770.1 | [^5^](#_ENREF_5) |
| *At*TFL1 | *Arabidopsis* | NP_196004.1 | [^4^](#_ENREF_4) |
| *At*BFT | *Arabidopsis* | NP_201010.1 | [^6^](#_ENREF_6) |
| *At*ATC | *Arabidopsis* | NP_180324.1 | [^7^](#_ENREF_7) |
| *At*MFT | *Arabidopsis* | NP_173250.1 | [^8^](#_ENREF_8) |
| FD | *Arabidopsis* | NP_195315.3 | [^9^](#_ENREF_9) |
| *AT*GRF2 | *Arabidopsis* | NP_565176.1 | [^10^](#_ENREF_10)^,^[^11^](#_ENREF_11) |
| *AT*GRF6 | *Arabidopsis* | NP_001190276.1 | [^11^](#_ENREF_11)^,^[^12^](#_ENREF_12) |
| *Os*Hd3a | *Oryza sativa* | BAB61028.1 | [^13^](#_ENREF_13) |
| *Os*RCN1 | *Oryza sativa* | XP_025876956.1 | [^14^](#_ENREF_14) |
| *Os*MFT | *Oryza sativa* | XP_015641734.1 | [^15^](#_ENREF_15) |
| *Os*FD1 | *Oryza sativa* | XP_015611345.1 | [^16^](#_ENREF_16) |
| *Os*FD2 | *Oryza sativa* | BAS99530.1 | [^16^](#_ENREF_16) |
| *Os*FD3 | *Oryza sativa* | XP_015623884.1 | [^16^](#_ENREF_16) |
| *Os*GF14b | *Oryza sativa* | XP_015635726.1 | [^16-18^](#_ENREF_16) |
| *Se*MFT1 | *Selaginella erythropus* | ACN54549.1 | [^15^](#_ENREF_15) |
| *Cs*MFT | *Camelina sativa* | XP_010476978.1 | [^15^](#_ENREF_15) |
| *Br*MFT | *Brassica rapa* | XP_009117626.2 |  |
| *Bo*MFT | *Brassica oleracea* | XP_013585084.1 |  |
| *Zj*MFT | *Ziziphus jujuba* | XP_015901015.1 |  |
| *Ch*FT | *Cardamine hirsuta* | AKC05615.1 |  |
| *Bs*FT | *Boechera stricta* | AIU56794.1 |  |
| *Bn*FT | *Brassica napus* | ACY03404.1 | [^4^](#_ENREF_4) |
| *Cs*TFL1 | *Camelina sativa* | XP_010423657.1 |  |
| *Aa*TFL1 | *Arabis alpina* | AEH43348.1 | [^19^](#_ENREF_19) |
| *Fi*TFL1 | *Fragaria iinumae* | AMS34800.1 | [^20^](#_ENREF_20) |
| *Sm*MFT1 | *Selaginella moellendorffii* | EFJ35292.1 | [^15^](#_ENREF_15) |
| *Sm*MFT2 | *Selaginella moellendorffii* | EFJ06396.1 | [^15^](#_ENREF_15) |
| *Sd* MFT1 | *Selaginella denticulata* | ACN54548.1 | [^15^](#_ENREF_15) |
| *Pe*FT | *Phyllostachys edulis* | JX141617.1 |  |
| *Pm*FT1 | *Phyllostachys meyeri* | AB498760.1 | [^3^](#_ENREF_3) |
| *Bt*FT | *Bambusa tulda* | KX290774.1 | [^2^](#_ENREF_2) |

# References

1 Biswas, P., Chakraborty, S., Dutta, S., Pal, A. & Das, M. Bamboo Flowering from the Perspective of Comparative Genomics and Transcriptomics. *Frontiers in plant science* **7**, 1900, doi:10.3389/fpls.2016.01900 (2016).

2 Dutta, S. *et al.* Identification, characterization and gene expression analyses of important flowering genes related to photoperiodic pathway in bamboo. *BMC genomics* **19**, 190, doi:10.1186/s12864-018-4571-7 (2018).

3 Hisamoto, Y. & Kobayashi, M. Flowering habit of two bamboo species,*Phyllostachys meyer i*and *Shibataea chinensis*, analyzed with flowering gene expression. *Plant Species Biology* **28**, 109-117, doi:10.1111/j.1442-1984.2012.00369.x (2013).

4 Karlgren, A. *et al.* Evolution of the PEBP gene family in plants: functional diversification in seed plant evolution. *Plant physiology* **156**, 1967-1977, doi:10.1104/pp.111.176206 (2011).

5 Yamaguchi, A., Kobayashi, Y., Goto, K., Abe, M. & Araki, T. TWIN SISTER OF FT (TSF) Acts as a Floral Pathway Integrator Redundantly with FT. *Plant & cell physiology* **46**, 1175-1189 (2005).

6 Yoo, S. J. *et al.* BROTHER OF FT AND TFL1 ( BFT ) has TFL1 -like activity and functions redundantly with TFL1 in inflorescence meristem development in Arabidopsis. *Plant Journal for Cell & Molecular Biology* **63**, 241–253 (2010).

7 Mimida, N. *et al.* Functional divergence of the TFL1‐like gene family in Arabidopsis revealed by characterization of a novel homologue. *Genes to Cells* **6**, 327-336 (2001).

8 Xi, W., Liu, C., Hou, X. & Yu, H. MOTHER OF FT AND TFL1 regulates seed germination through a negative feedback loop modulating ABA signaling in Arabidopsis. *The Plant cell* **22**, 1733-1748, doi:10.1105/tpc.109.073072 (2010).

9 Abe, M. *et al.* FD, a bZIP Protein Mediating Signals from the Floral Pathway Integrator FT at the Shoot Apex. *Science (New York, N.Y.)* **309**, 1052 (2005).

10 Nakkaew, A., Thitichai, N., Nualkaew, S., Chotigeat, W. & Phongdara, A. Cloning, characterization and overexpression of a 14‐3‐3 ω protein from oil palm (*E laeis guineensis*). *Plant Breeding* **132**, 701-710 (2013).

11 Fulgosi, H. *et al.* 14-3-3 proteins and plant development. *Plant molecular biology* **50**, 1019-1029 (2002).

12 Zhao, Y. *et al.* Analysis and Prediction of QL14h by Database Application. *Wireless Personal Communications* **103**, 585-593 (2018).

13 Kojima, S. *et al.* Hd3a, a rice ortholog of the Arabidopsis FT gene, promotes transition to flowering downstream of Hd1 under short-day conditions. *Plant & cell physiology* **43**, 1096-1105 (2002).

14 M, N., K, S. & J, K. Overexpression of RCN1 and RCN2, rice TERMINAL FLOWER 1/CENTRORADIALIS homologs, confers delay of phase transition and altered panicle morphology in rice. *Plant Journal* **29**, 743-750 (2002).

15 Hedman, H., Kallman, T. & Lagercrantz, U. Early evolution of the MFT-like gene family in plants. *Plant molecular biology* **70**, 359-369, doi:10.1007/s11103-009-9478-x (2009).

16 Meng, X., Muszynski, M. G. & Danilevskaya, O. N. The FT-like ZCN8 gene functions as a floral activator and is involved in photoperiod sensitivity in maize. *The Plant cell* **23**, 942-960 (2011).

17 Taoka, K. *et al.* 14-3-3 proteins act as intracellular receptors for rice Hd3a florigen. *Nature* **476**, 332-335, doi:10.1038/nature10272 (2011).

18 Purwestri, Y. A., Ogaki, Y., Tamaki, S., Tsuji, H. & Shimamoto, K. The 14-3-3 protein GF14c acts as a negative regulator of flowering in rice by interacting with the florigen Hd3a. *Plant and cell physiology* **50**, 429-438 (2009).

19 Wang, R. *et al.* Aa TFL1 confers an age-dependent response to vernalization in perennial Arabis alpina. *The Plant cell* **23**, 1307-1321 (2011).

20 Koskela, E. A. *et al.* TERMINAL FLOWER 1 is a breeding target for a novel everbearing trait and tailored flowering responses in cultivated strawberry (*Fragaria× ananassa Duch.*). *Plant biotechnology journal* **14**, 1852-1861 (2016).
